# Unlocking the genomic potential of historical and formalin-fixed specimens: phylogenetic insights from museum-preserved threadfin fishes (Teleostei: Polynemidae)

**DOI:** 10.7717/peerj.20029

**Published:** 2025-09-30

**Authors:** Matthew G. Girard, Kevin R. Chovanec

**Affiliations:** 1 Vertebrate Zoology, National Museum of Natural History, Smithsonian Institution, Washington, DC, United States of America; 2 Biodiversity Institute, University of Kansas, Lawrence, KS, United States of America

**Keywords:** Combined analyses, Mitochondrial genome, *Polydactylus*, Ultraconserved elements, UCEs

## Abstract

DNA sequencing continues to revolutionize our understanding of biodiversity, ecology, and evolution. While analyzing sequence data allows us to address countless questions, most of the world’s vertebrate museum specimens have been historically inaccessible for genetic sampling. This is partially due to the absence of modern genetic samples and/or the impact formalin has on DNA during the preservation of specimen vouchers. Recent studies have shown successful extraction of DNA from historic museum specimens using additional chemicals and/or exposing the sample(s) to heat, with these advances enhancing the possibility of capturing genomic information from type specimens, characterizing genetic diversity within species complexes, and incorporating rare samples into phylogenetic analyses. However, questions remain about the reliability of these data and utility of historic DNA in modern phylogenetic analyses. In this study, we use a commercial extraction kit that targets formalin-fixed, paraffin-embedded samples to successfully extract DNA from historic museum specimens of threadfin fishes (Teleostei: Polynemidae). These specimens represent rare, genetically uncharacterized taxa that have yet to be included in a phylogenetic analysis. Low-depth shotgun sequencing is then used to sequence mitochondrial loci from the historic samples. The resulting sequence data are assembled, validated, and incorporated into a newly generated mitochondrial dataset that is simultaneously analyzed with a previously published ultraconserved-element dataset to construct a phylogenetic framework. We then explore new and previously described morphological variation within this new evolutionary framework for threadfins, identifying several shared characters that warrant revision of the generic-level classification. These findings add to the growing body of literature that demonstrates sequencing historical DNA from museum specimens and analyzing these data with complementary datasets of molecular markers from modern genetic samples can provide reliable and comprehensive assessments of biodiversity, ecology, and evolution across fishes and other vertebrates.

## Introduction

Molecular characters have provided a wealth of information about the planet’s biodiversity. Since the development of standardized primers to amplify mitochondrial loci (*e.g.*, Kocher et al., 1989), researchers have employed DNA barcoding to assess biodiversity (*e.g.*, Hebert et al., 2003), metagenomics to track ecosystem change (*e.g.*, Stein et al., 1996), and DNA-based phylogenetics to gain insights into evolutionary history. Within vertebrates, DNA-based phylogenetics has enabled researchers to identify novel hypotheses of relationships in amphibians and reptiles (*e.g.*, Feller & Hedges, 1998; Hedges & Poling, 1999), birds (*e.g.*, Edwards & Wilson, 1990; Edwards, Arctander & Wilson, 1991), fishes (*e.g.*, Chen, Bonillo & Lecointre, 2003; Miya et al., 2003), and mammals (*e.g.*, Madsen et al., 2001; Murphy et al., 2001). As new molecular techniques emerged, the questions posed simultaneously expanded in scope and scale, leading to the development of datasets that included more loci and phylogenies that encompassed a broader range of taxa (*e.g.*, Smith & Craig, 2007; Gardner et al., 2010; Pyron & Wiens, 2011; Near et al., 2012; Near et al., 2013; Betancur et al., 2017; Foley et al., 2023; Stiller et al., 2024). These datasets and analyses continue to contribute to our understanding of the intra- and interrelationships of vertebrate groups and address questions about large-scale evolutionary patterns (*e.g.*, Alfaro et al., 2018; Rabosky et al., 2018; Ghezelayagh et al., 2022; Christmas et al., 2023). While genomic data continues to have a profound impact, these data have generally been sequenced from recently collected specimens that have associated genetic samples. Many species lack modern genomic resources, leaving numerous questions about biodiversity, ecology, and evolution unanswered. Hereafter, the usage of “modern” for genetic samples and other genomic resources applies to samples that were purposefully collected for genetic analyses (*i.e.,* sampled prior to specimen fixation and preserved in ethanol, liquid nitrogen, or similar).

Natural history museums house millions of preserved specimens representing the diversity of taxa from across the globe, including abundant, rare, described, and undescribed species. These libraries of life document our pursuit to understand the biodiversity of our planet and hold the answers to countless questions within biodiversity sciences. Early attempts to extract DNA from fluid-preserved museum specimens yielded mixed success (*e.g.*, Chakraborty, Sakai & Iwatsuki, 2006; Zhang, 2010), often due to the heavy crosslinking that occurs between strands of DNA during formalin fixation. Degradation and fragmentation of historic DNA (hDNA) can also impact the accuracy of base calls in the resulting reads, yielding unreliable results (*e.g.*, Cooper, Smith & Westneat, 2009, but see Bernardi, 2011). Recent studies have shown promising results for hDNA extraction using various methods and technological improvements (*e.g.*, Ruane & Austin, 2017; Silva et al., 2019; Appleyard et al., 2022; Hawkins et al., 2022; Sullivan et al., 2022; Speer et al., 2022), including sampling DNA-rich liver samples (*e.g.*, Hykin, Bi & McGuire, 2015; Zacho et al., 2021; Hahn et al., 2022), exposing samples to elevated temperatures for various amounts of time to remove crosslinking (*e.g.*,  Straube et al., 2021), or lysing samples in alkaline environments to enhance enzymatic digestion (*e.g.*, Hahn et al., 2024). Some of these new techniques can be found within commercially available extraction kits targeting formalin-fixed, paraffin-embedded (FFPE) samples (*e.g.*, exposing samples to elevated temperatures), and commercially produced single-stranded library preparation kits are increasingly available for the conversion of short and damaged DNA. These advances in extraction and library preparation are allowing researchers to sequence genomic information from type specimens, characterize genetic diversity within species complexes, and incorporate rare samples into phylogenetic analyses (*e.g.*, Sullivan et al., 2022; Bernstein et al., 2023; Quattrini et al., 2024; Muschick, Rüber & Matschiner, 2025).

Commonly called threadfins for their elongate and thread-like pectoral-fin rays, the fish family Polynemidae includes eight genera, with 21 of the 42 species placed in a single genus, *Polydactylus*. Four previous studies have targeted the intrarelationships of this family, with three analyzing morphological characters (*i.e.,* Feltes, 1986; Kang, 2017; Presti, Johnson & Datovo, 2023) and one (Girard et al., 2022a) analyzing a combination of mitochondrial and ultraconserved element (UCE) loci. While these studies have sampled between 19 (Presti, Johnson & Datovo, 2023) and 30 species (Girard et al., 2022a), recovering different relationships among them, all have consistently recovered the genus *Polydactylus* as polyphyletic. Hereafter, species of *Polydactylus* that are recovered outside of the monophyletic group that includes the type species of the genus (*i.e.,* *P. virginicus*) are listed as “*Polydactylus.*” Girard et al. (2022a) proposed names for several clades of “*Polydactylus*” (*i.e.,* black-spotted species, striped species) but did not revise the classification or the taxonomy of the genus, as multiple key taxa were missing from their analyses due to a lack of modern genetic samples. Some of these unsampled species have been allied with groups of “*Polydactylus*” in previous taxonomic works (*e.g.*, “*P.*” *persicus* with black-spotted species, Motomura & Iwatsuki, 2001b). Other key taxa have morphological characters that could ally them with several species groups within the family, such as the highly elongate threadlike pectoral-fin rays in “*P.*” *macrophthalmus*. Highly elongate pectoral-fin rays are found in several species of threadfins in at least three genera, including *Filimanus,* “*Polydactylus*,” and all species of *Polynemus* (Motomura, 2004) making the placement of “*P.*” *macrophthalmus* difficult based on morphology alone. This and other unsampled species of “*Polydactylus*” are ideal targets for new hDNA techniques because they lack modern genetic resources, they have yet to be included in any phylogenetic analyses, and their confusing suites of anatomical characters obfuscate their phylogenetic placement within the family when analyzing morphology alone.

To build on the advances in hDNA extraction and library preparation, leverage the ease of commercially available FFPE extraction and single-stranded library preparation kits, and test the utility of hDNA data in modern phylogenetic analyses, we targeted two enigmatic species of “*Polydactylus*” for hDNA sampling. We successfully extracted and sequenced mitochondrial loci from the livers of two historical museum specimens representing “*P.*” *bifurcus* and “*P.*” *macrophthalmus*. As previous studies have shown, analyzing datasets of mitochondrial loci alongside complementary multigene nuclear datasets of representative taxa can produce robust phylogenetic hypotheses both within polynemids (Girard et al., 2022a) and other groups of animals (*e.g.*, Talavera et al., 2021). We apply a similar integrative approach, analyzing the hDNA mitochondrial loci alongside a novel mitochondrial dataset and the ultraconserved elements (UCE) dataset published by Girard et al. (2022a) to generate a hypothesis of relationships. Given the recovered placement of the hDNA-sampled taxa, we explore new and previously described morphological variation within threadfins, highlighting several shared characters that support the placement of these taxa with their recovered sister groups. With these newly identified synapomorphies, the taxonomy of the family is revised, including the description of a new genus.

## Materials & Methods

**Figure 1:**
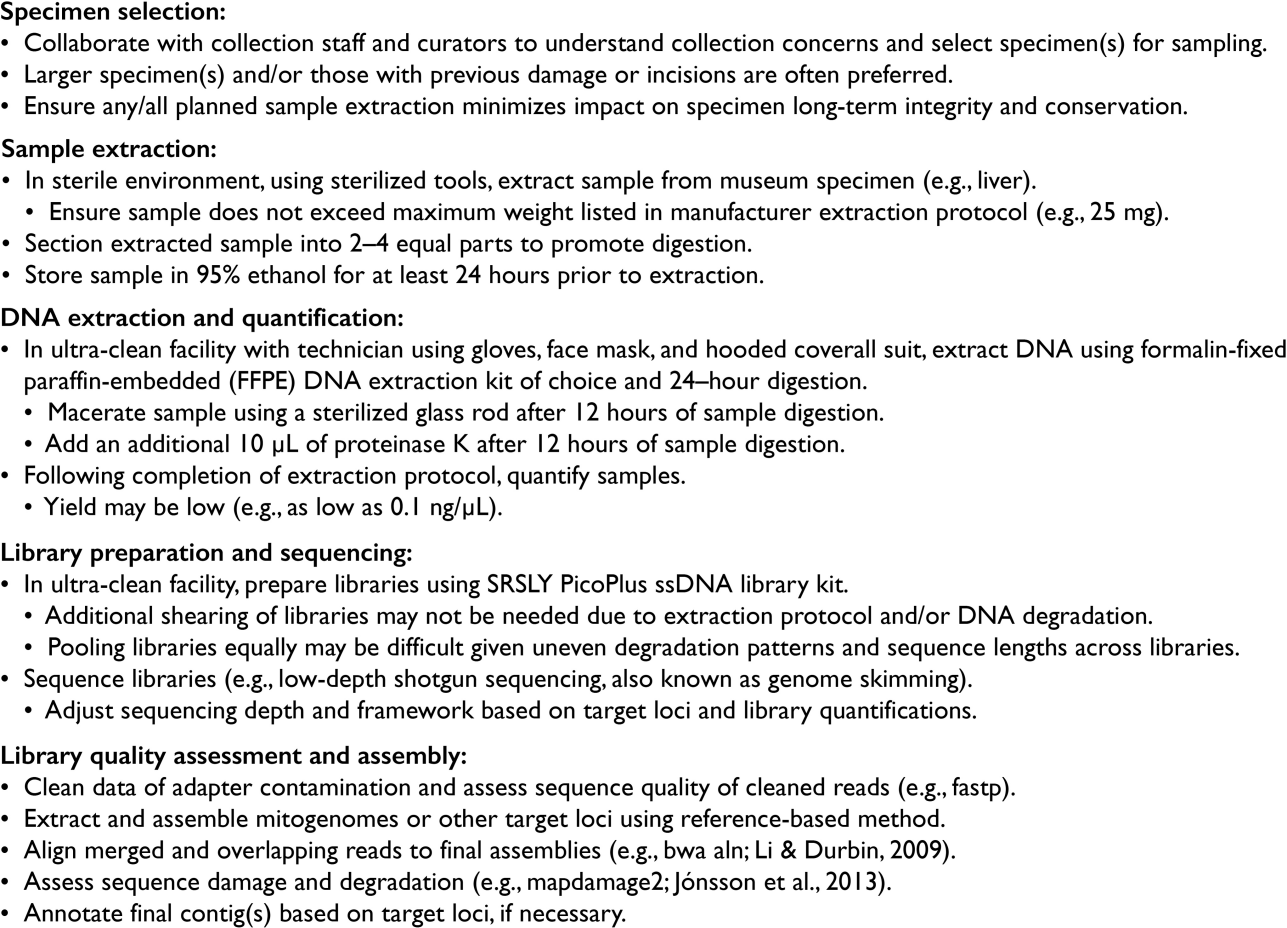
Generalized workflow for sequencing, assembling, and validating DNA from historic museum specimens used in this study. Additonal information and specifics relating to this study can be found in the Materials & Methods section.

All taxa used in this study, along with associated museum catalog information, can be found in File S1, with codes for museum collections following Sabaj (2020). A visualization of sampling, extraction, library preparation, and sequencing protocols used in this study is shown in Fig. 1.

### Sampling, extraction, and sequencing of DNA from historic samples

The specimen of “*P.*” *bifurcus* (USNM 76627; 125 mm standard length [SL]) was collected by F. Baker in Kao-Hsiung, Taiwan, on 3–4 December 1914. The specimen has been stored in 70–75% ethanol and lacks records for methods of fixation. The specimen of “*P.*” *macrophthalmus* (ZRC 39003; 208 mm SL) was collected by H. H. Ng and colleagues at a market in Jambi, southern Sumatra, Indonesia, in June 1995. The specimen was fixed in 4% formalin for ∼3 weeks prior to transfer to ∼70% ethanol for long-term storage. Samples of liver were dissected from each of these specimens through an abdominal incision. Dissections occurred in sterile environments, using sterilized scalpels and forceps. Liver was chosen for extraction due to its higher DNA yield among formalin-fixed samples (*e.g.*, Hahn et al., 2022). Liver samples weighing between 13 (“*P.*” *bifurcus*) and 24 mg (“*P.*” *macrophthalmus*) were divided into 2–4 equal parts after dissection and stored in two mL tubes of 95% ethanol for at least 24 h prior to hDNA extraction. Extractions were performed in the Historic DNA Laboratory at the Laboratories of Analytical Biology (Smithsonian Institution) with the individual performing the extraction mitigating contamination by wearing gloves, a face mask, a hair net, and a hooded coverall suit. Samples were extracted using a Quick-DNA/RNA FFPE Miniprep Kit (Zymo Research) following manufacturer’s extraction protocols for “Total Nucleic Acid Purification,” with the following modifications: omitting the deparaffinization preparation step; macerating the sample using a sterilized glass rod after 12 h of the 24-hour sample-digestion step; adding 10 µL of proteinase K after 12 h of the 24-hour sample-digestion step. Extracted samples were quantified using 2 µL of the hDNA extract and the dsDNA HR Assay Kit (Thermo Fisher Scientific) with Qubit Fluorometer (Thermo Fisher Scientific). Final 98 µL samples were sent to Daicel Arbor Biosciences for library preparation using the SRSLY PicoPlus ssDNA library kit (Claret Bioscience), sequencing, and demultiplexing. Libraries were sequenced using a low-depth whole-genome-sequencing approach commonly applied in genome skimming (*e.g.*, Hoban et al., 2022; Quattrini et al., 2024). This sequencing approach was chosen as it yields reads for many loci while bypassing the complications of amplifying target regions of the genome from degraded samples. Further, mitochondrial loci have been shown to be abundant in modern DNA samples that were genome skimmed. Mitochondrial locus lengths, arrangements, and start and stop codons have been well studied in vertebrates (*e.g.*, Miya et al., 2003), making orthologous loci from congener taxa readily available for comparison to hDNA samples. Samples were sequenced in a 150-base-pair (bp) paired-end framework on a NovaSeq 6000 (Illumina) targeting 20 million reads per sample. Sample extraction, library quantification, and sequencing results can be found in Table 1.

**Table 1:** Sequencing statistics and GenBank information for genome-skimmed samples.

**Family**	**Species**	**Type of genetic sample**	**Concentration of DNA after extraction (ng/μ L)**	**Concentration of DNA after library prep (ng/μ L)**	**Total clean reads sequenced**	**GC% clean reads sequenced**	**Reference mitogenome used for mapping**	**Reads mapped to reference mitogenome**	**Percentage of clean reads mapped**	**Annotated mitogenome length**	**Mean coverage depth (×)**
Mugilidae	*Mugil curema*	Modern DNA	0.159	2.1	12,797,874	41	KP018403	51,464	0.40%	16,828	292.4
Polynemidae	*Filimanus perplexa*	Modern DNA	2.688	34.9	16,226,057	40	PV590078	94,009	0.58%	17,117	457.4
Polynemidae	*Filimanus xanthonema*	Modern DNA	0.305	1.6	13,948,392	41	PV590078	159,487	1.14%	17,428	855.9
Polynemidae	*Filistriatus bifurcus* gen. nov.	Historic DNA	0.355	9.5	37,034,349	46	NC_026235	22,057^*^	0.06%^*^	16,564^*^	111.2^*^
Polynemidae	*Galeoides decadactylus*	Modern DNA	20.466	40.9	24,947,149	40	NC_027088	49,144	0.20%	16,804	283.1
Polynemidae	*Leptomelanosoma macrophthalmus*	Historic DNA	0.118	28.9	86,551,031	43	PV590078	13,622^*^	0.01%^*^	16,346^*^	62.9^*^
Polynemidae	*Parapolynemus verekeri*	Modern DNA	5.881	52.9	16,256,888	40	NC_026236	20,426	0.13%	16,698	101.3
Polynemidae	*Pentanemus quinquarius*	Modern DNA	13.845	55.4	22,943,533	41	NC_057649	61,636	0.27%	16,681	315.1
Polynemidae	*Polydactylus approximans*	Modern DNA	8.181	49.1	21,701,244	41	OP056931	11,583	0.05%	16,713	51.0
Polynemidae	*“Polydactylus” macrochir*	Modern DNA	3.467	45.1	13,887,832	41	MW630081	15,028	0.11%	16,670	56.3
Polynemidae	*“Polydactylus” multiradiatus*	Modern DNA	0.83	10.8	12,758,097	43	NC_026235	19,231	0.15%	16,711	102.6
Polynemidae	*“Polydactylus” nigripinnis*	Modern DNA	6.31	50.5	22,426,340	41	PV590087	11,259	0.05%	16,683	53.7
Polynemidae	*Polydactylus octonemus*	Modern DNA	5.985	47.9	17,041,943	41	OP056931	49,929	0.29%	16,665	250.0
Polynemidae	*Polydactylus opercularis*	Modern DNA	8.353	50.1	21,888,387	40	MW630081	48,432	0.22%	16,606	257.9
Polynemidae	*“Polydactylus” quadrifilis*	Modern DNA	9.83	49.2	20,401,301	39	PV590078	55,678	0.27%	17,108	244.2
Polynemidae	*Polynemus melanochir*	Modern DNA	2.195	28.5	17,134,260	40	NC_026236	31,514	0.18%	16,834	143.7
Polynemidae	*Polynemus multifilis*	Modern DNA	2.835	36.8	20,976,546	40	NC_026236	43,712	0.21%	16,848	217.5

**Notes.**

Information for mitochondrial genomes extracted from SRAs not included in table (see File S1).

* indicate values after bwa aln and deduplication.

### Sampling, extraction, and sequencing of DNA from modern genetic samples

To test the utility of hDNA in phylogenetic analyses, we analyzed mitochondrial and nuclear loci in combination to generate a phylogeny (*e.g.*, Martin et al., 2018; Talavera et al., 2021; Smith et al., 2022; Smith et al., 2024). Taxon sampling was primarily based on the dataset published by Girard et al. (2022a), as their study includes the greatest number of threadfin species to date. We used the ‘32 terminal’ UCE dataset in Girard et al. (2022a) that included 20 species of polynemids (∼47% family diversity) and 12 outgroup taxa. A complementary mitochondrial dataset was generated in this study that sampled all taxa represented in the ‘32 terminal’ dataset as well as an additional 10 species of threadfins. In total, the combined datasets sample 32 of 42 species (≈76%) of threadfins and 12 outgroup taxa (File S1). Outgroup taxa include representatives from the Bedotiidae, Centrarchidae, Latidae, Mugilidae, Pleuronectidae, Psettodidae, Sciaenidae, Scombridae, Scophthalmidae, and Sphyraenidae. DNA was extracted from modern genetic samples using an AutoGenPrep 965 automated DNA extraction robot, following the manufacturer’s protocols. Libraries were prepared using the NEB Ultra II FS DNA library prep kit (New England Biolabs), following the manufacturer’s protocol, and iTru y-yoke adapter and dual indices (Glenn et al., 2019). Libraries were quantified using the fluorometer and assay kit listed above and pooled for sequencing. Extractions and library preparation were performed in the Laboratories of Analytical Biology (Smithsonian Institution). Pooled libraries were sent to Oklahoma Medical Research Foundation NGS Core for sequencing using a NovaSeq 6000 (Illumina) and a paired-end 150 bp framework targeting 13 million reads per sample. Sample extraction, library quantification, and sequencing results can be found in Table 1.

### Mitogenome assembly, annotation, and quality assessment

**Figure 2:**
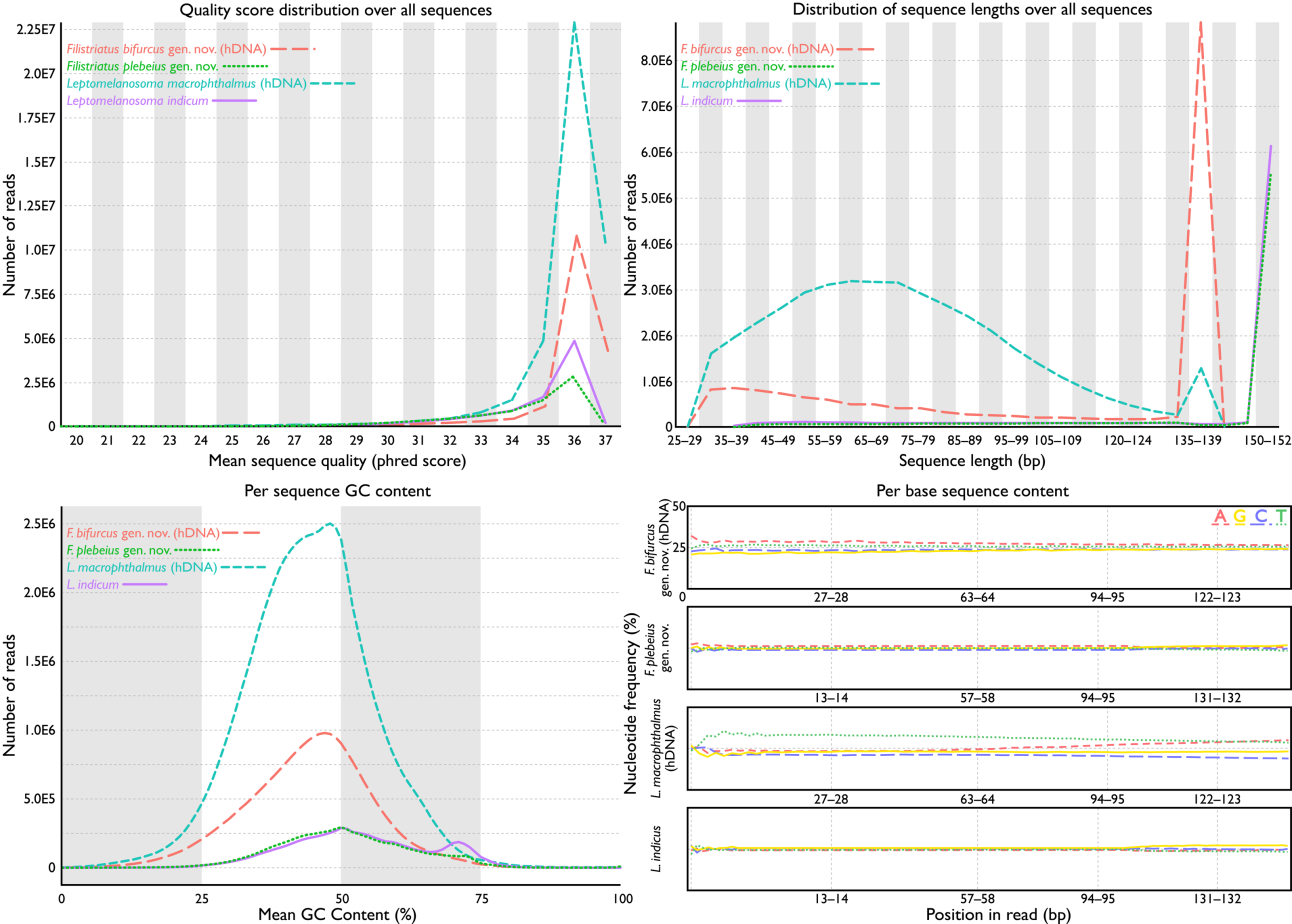
FastQC assessments of sequence quality for historic *versus* modern DNA. Taxa selected for comparison based on phylogenetic relatedness (see phylogeny). Historic DNA libraries were prepared for genome skimming. Modern DNA libraries were prepared using target-capture approach. Modern DNA libraries prepared for genome skimming are similar in quality score distributions, per-sequence GC content, and per-base sequence content to modern DNA libraries prepared for target capture. Distribution of sequence lengths were more variable for genome-skimmed libraries than target-capture libraries.

Demultiplexed sequence data from multiple runs were received in compressed FASTQ format. These data were uncompressed into two read files per taxon and uploaded to GenBank (SRA Accession Numbers [SRR33326234 –SRR33326248 ]; BioProject PRJNA720393; File S1). The data were cleaned of adapter contamination using fastp (Chen et al., 2018) and FastQC version 0.12.1 (Babraham Bioinformatics) was used to assess sequence quality (Fig. 2). Cleaned reads were collated with previously published SRA data obtained from GenBank for assembly (BioProjects PRJNA341709, PRJNA604383, PRJNA720393, PRJNA796495; File S1). A reference-based method within Geneious version 11.1.5 (Kearse et al., 2012) was used to assemble reads into mitochondrial contigs. Multiple reference sequences were tested for assembly, with the putative closest threadfin taxon based on the phylogeny presented in Girard et al. (2022a; if available) generally used as the reference sequence for assembly (see Table 1). The ‘map to reference’ function in Geneious was set to ‘medium-low sensitivity’ and ‘iterate up to five times.’ To assess DNA damage in historic samples, we aligned merged, overlapping read pairs to final assemblies using bwa aln (Li & Durbin, 2009), removed duplicates and reads with MAPQ scores <20, and analyzed resulting bam files with mapdamage2 (Jónsson et al., 2013). Historical and modern mitogenomes were then annotated using MitoAnnotator (Iwasaki et al., 2013; Sato et al., 2018; Zhu et al., 2023). Annotated mitogenomes were submitted to GenBank and assigned accession numbers (PV590073 –PV590096; PV590343 –PV590344; PV593525; see File S1).

### Partitioning schemes and phylogenetic analyses

For the UCE dataset, we used the same partitioning scheme of 555 subsets and associated models as listed in Girard et al. (2022a). For the mitochondrial dataset, orthologous loci for two rRNA and 13 protein-coding regions of sequenced mitogenomes were collated into individual FASTA files and aligned with MAFFT version 7 (Katoh & Standley, 2013). Additionally, COI ‘barcode’ sequences from *Filimanus simils* (MF281368), “*P.*” *longipes* (PV590860), “*P.*” *mullani* (MF281374), and *P. oligodon* (JQ365495) were included in the COI alignment (as in Girard et al., 2022a). Lengths of alignments were as follows, with completeness at the level of individual bp in parentheses: 12S 1,050 bp (89%); 16S 1,900 bp (88%); ATPase6 702 bp (96%); ATPase8 189 bps (88%); COI 1,566 bp (93%); COII 695 bp (99%); COIII 786 bp (96%); CytB 1,165 bp (96%); ND1 978 bp (99%); ND2 1,102 bp (93%); ND3 349 bp (100%); ND4 1,385 bp (98%); ND4L 297 bp (100%); ND5 1,872 bp (97%); ND6 536 bp (98%). Individual matrices were concatenated for partitioning and phylogenetic inference. The final alignment was 14,572 bp in length and 89% complete at the level of individual bp. IQ-TREE version 2.2.2.6 (TESTMERGEONLY with rclusterf 50; Chernomor, Von Haeseler & Minh, 2016; Kalyaanamoorthy et al., 2017; Minh et al., 2020) recovered an optimal partitioning scheme of 12 partitions and associated models. Model assessment and resulting partition information can be found in Files S2 and S3. The mitochondrial dataset, UCE dataset, and associated partitioning schemes were analyzed simultaneously using IQ-TREE. Twenty tree searches were performed with the perturbation strength (-pers) set to 0.2 and the number of unsuccessful iterations to stop (-nstop) set to 2,000. Support for the best-fitting topology was generated using 500 standard bootstrap replicates (-bo) and reconciled with the most likely phylogeny using IQ-TREE (-con; Files S4 and S5).

### Morphological investigation

We explored new and previously described (*e.g.*, Marathe & Bal, 1958; Kang, 2017; Presti, Johnson & Datovo, 2023) morphological variation as they related to the placement of “*P.*” *bifurcus* and “*P.*” *macrophthalmus* in the phylogeny. Microcomputed tomography (µCT) was used to examine internal osteology of museum specimens. Specimens were scanned using a GE Phoenix v—tome— x M 240/180 kV Dual Tube µCT at the National Museum of Natural History, Smithsonian Institution. Scan settings were 90–130 kV, 130–190 µA, 250–500 ms exposure time, and 25–57 µm voxel size. Resulting scans are available through MorphoSource project ID [000735880] and media identifiers (*L*. *indicum* USNM 357761 [000735950], *Pentanemus quinquarius* UF 221610 [000167767], “*P.*” *bifurcus* USNM 76627 [000735955], “*Polydactylus*” *macrophthalmus* UMMZ 171714 [000735937], “*P*.” *plebeius* USNM 403223 [000735960], “*P*.” *sexfilis* CSIRO C261 [000735945], and *P*. *virginicus* FMNH 104648 [000735940]). All scan data were visualized and segmented using the SlicerMorph module (Rolfe et al., 2021) in 3D Slicer (Fedorov et al., 2012) and the protocol described in Girard et al. (2022b). All other specimen imaging was performed using equipment and protocols listed in Girard, Davis & Smith (2020).

### Nomenclature

The electronic version of this article in Portable Document Format (PDF) will represent a published work according to the International Commission on Zoological Nomenclature (ICZN), and hence the new names contained in the electronic version are effectively published under that Code from the electronic edition alone. This published work and the nomenclatural acts it contains have been registered in ZooBank, the online registration system for the ICZN. The ZooBank LSIDs (Life Science Identifiers) can be resolved and the associated information viewed through any standard web browser by appending the LSID to the prefix http://zoobank.org/. The LSID for this publication is: urn:lsid:zoobank.org:pub:E0E70F44-3417-4BD9-87EF-F4F2CD157F68. The online version of this work is archived and available from the following digital repositories: *PeerJ*, PubMed Central SCIE and CLOCKSS.

## Results

### Assessment and annotation of sequencing reads

Total cleaned reads generated from hDNA samples of “*P.*” *bifurcus* and “*P.*” *macrophthalmus* equaled 37,034,349 and 86,551,031 bp, respectively. Mean number of reads from modern genetic samples equaled 18,834,790 bp (range: 12,758,097–24,947,149 bp; see Table 1). Analyses of reads *via* FastQC showed similarly acceptable per-base sequence quality assessments, per-sequence quality scores, per-base N content, and sequence duplication levels across all samples (Fig. 2). FastQC flagged GC content for 18 of the 27 samples. These flags were generally associated with samples where the percent GC content was under 42. Mean percent GC content across all samples was 41, with a range of 39–46 (Table 1). Distribution of sequence lengths was more variable from libraries prepared for genome skimming than those for target capture, with a greater abundance of shorter reads in the genome-skimmed libraries. The greatest variability in sequencing-read length came from the hDNA sample of “*P.*” *macrophthalmus* (Fig. 1).

**Figure 3:**
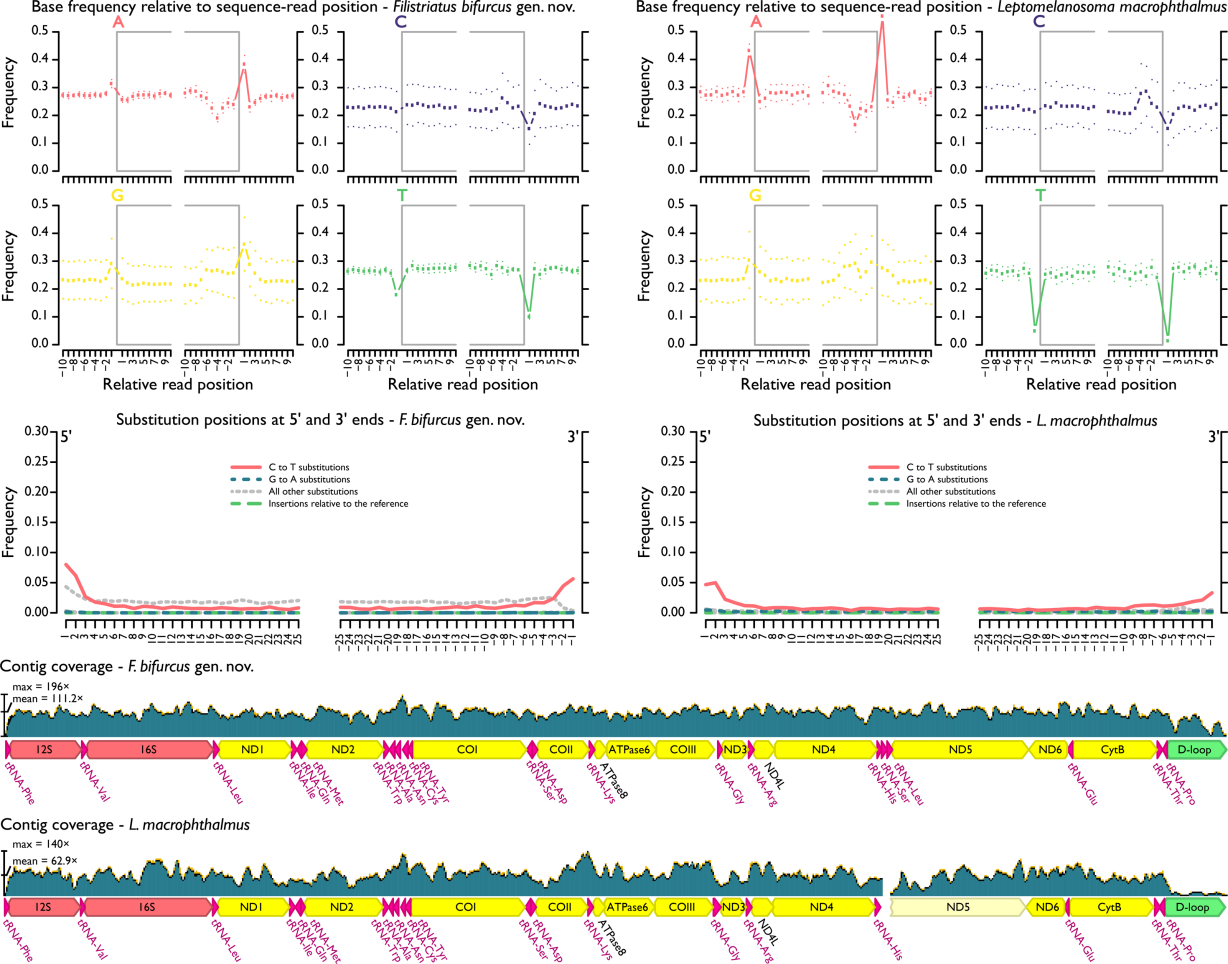
MapDamage2 assessments of base pairs and contig coverage for historic DNA. Upper plots highlight position and frequency of nucleotides both within and outside the sequence read, with grey boxes corresponding to the read. Strong deviation in base-call frequency immediately outside of the read indicates read damage. Middle plots highlight position of specific substitutions from the 5^′^ and the 3^′^ ends. Overall, misincorporations followed general trends found in other studies (*e.g.*, Dabney, Meyer & Pääbo, 2013) and were concentrated at the ends of sequencing reads. Lower plots show distribution of coverage across hDNA contigs. Additional files output from mapDamage2 not shown here are included in the supplement.

Assembled and adjusted contigs from hDNA samples were 16,346 (“*P.*” *macrophthalmus*) and 16,564 bp (“*P.*” *bifurcus*). After quality filtering and duplicate removal, mean contig coverage range was 63.9–111.2× and mean fragment length range was 77.3–83.5 bp (Fig. 3, Table 1). As expected (*e.g.*, Dabney, Meyer & Pääbo, 2013), terminal deamination was found on the 5′ and 3′ ends of hDNA reads, with deamination frequencies ranging between 5% and 8% in “*P.*” *macrophthalmus* and “*P.*” *bifurcus*, respectively (see Files S6).

**Figure 4:**
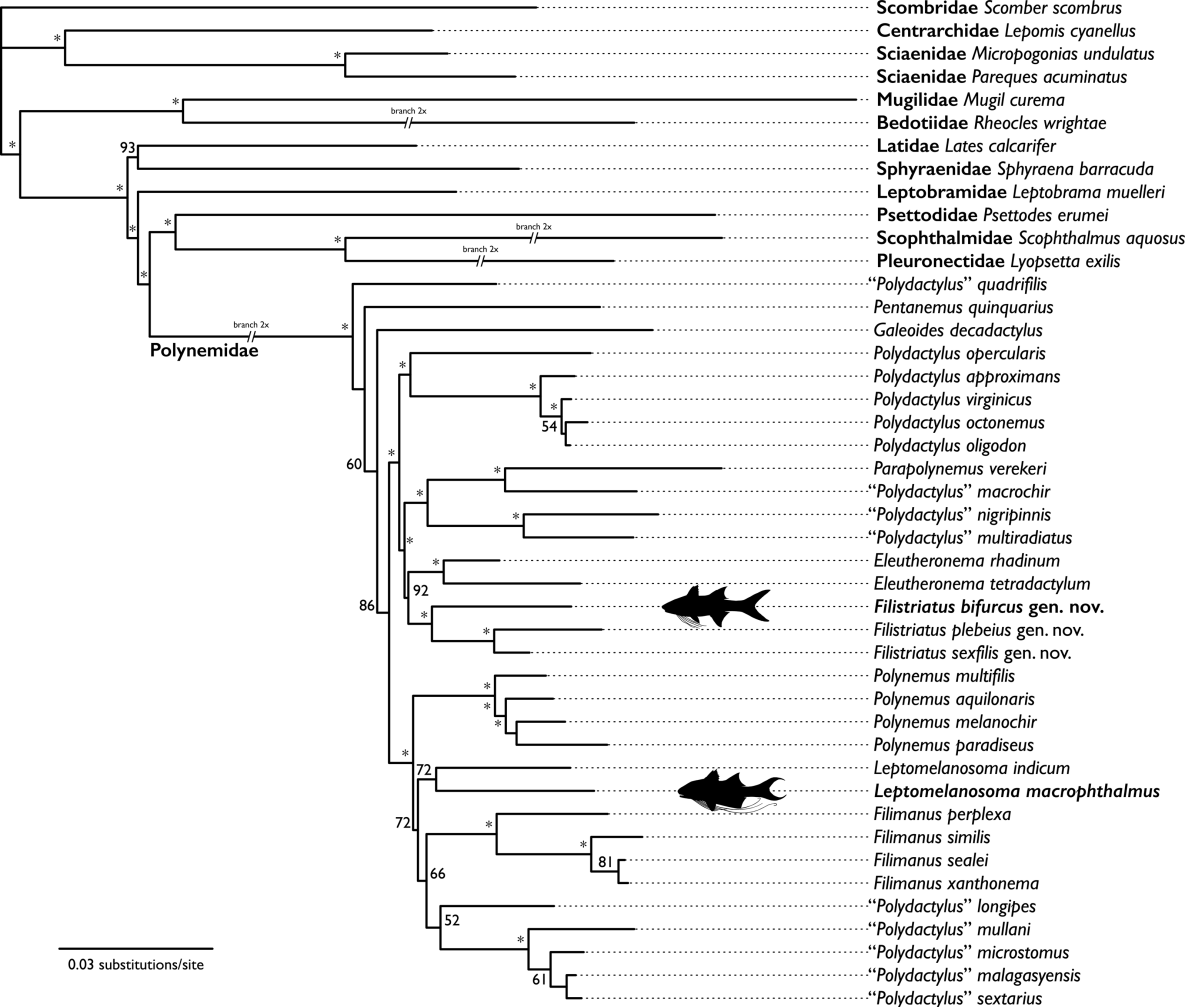
Hypothesis of relationships from likelihood analysis of polynemid and outgroup taxa based on mitochondrial and UCE datasets. Bootstrap support values <50 not shown. Bootstrap support values indicated by “*” indicate support value ≥95. Taxa with representative silhouettes highlight samples sequenced from historic museum specimens.

Mitogenomes for 23 species of threadfins were assembled and annotated from newly sequenced modern samples and previously sequenced SRAs. Contig lengths ranged from 16,606–17,428 bps, with mean contig coverage ranging from 51–855.9× (Table 1). Across modern and historic samples, all polynemid mitogenomes encoded 13 protein-coding loci, two rRNAs, and one D-loop. All but one sample encoded 22 tRNAs (tRNA-Ser and tRNA-Leu removed from “*P.*” *macrophthalmus*). Locus orientation and order match those found in previously sequenced species of the Polynemidae (Miya et al., 2003). For two of the outgroup taxa in the dataset that did not have mitogenomes publicly available (Files S1), only a subset of mitochondrial loci were able to be extracted from previously published SRAs sequenced from modern genetic samples. These include: *Leptobrama muelleri* (2 rRNA, 11 protein-coding loci) and *Rheocles wrightae* (1 rRNA, 8 protein-coding loci; Files S1).

### Phylogenetic analyses

The hypothesis of relationships recovered from the analysis is shown in Fig. 4. The bootstrap analysis yielded 32 nodes (of 40; ≈80%) with a value of ≥80% and 28 nodes (≈70%) with a value of ≥95% (Fig. 4). All nodes outside of the Polynemidae were supported with ≥93% bootstrap support. Within the Polynemidae, bootstrap supports were higher than those found by Girard et al. (2022a) in their combined analysis. This is likely due to this analysis sampling multiple mitochondrial loci *versus* only one mitochondrial locus as in Girard et al. (2022a).

The resulting topology showed a similar topology to that of Girard et al. (2022a) except for “*Polydactylus*” *quadrifilis*, *Pentanemus quinquarius*, and *Galeoides decadactylus* being recovered as an early diverging grade sister to the rest of the Polynemidae rather than a clade. “*Polydactylus*” *macrophthalmus* is recovered sister to *Leptomelanosoma indicum*. “*Polydactylus*” *bifurcus* is recovered sister to a clade of “*P.*” *plebeius* and “*P.*” *sexfilis*. Given the non-monophyly of *Polydactylus* in multiple studies (*e.g.*, Kang, 2017; Girard et al., 2022a; Presti, Johnson & Datovo, 2023) and the type species of the genus (*P. virginicus*) recovered in a distant clade, we describe a new genus for “*P.*” *bifurcus,* “*P.*” *plebeius*, and “*P.*” *sexfilis* and reclassify “*P.*” *macrophthalmus* in the genus *Leptomelanosoma*.

### Taxonomic modifications to the Polynemidae

***Filistriatus*** **gen. nov. Girard** urn:lsid:zoobank.org:act:983362F8-2F4E-4730-A9BE-BE24F2784474

Diagnosis: a genus of small-to-moderately-sized threadfins differentiated from all other genera of the Polynemidae by the following combination of characters: 7–9 dark stripes along the longitudinal scale rows above the lateral line, 7–9 faint stripes along the longitudinal scale rows below the lateral line, reduced lateral pore of the supraorbital canal, reduced sensory canal commissure on the dorsal surface of the frontal, 5–6 free pectoral-fin rays, and 54–72 lateral-line scales.

Type species: *Polynemus sexfilis* Valenciennes 1831

Included species: *Polydactylus bifurcus* Motomura, Kimura & Iwatsuki 2001, *Polydactylus plebeius* (Broussonet 1782), *Polydactylus sexfilis* (Valenciennes 1831), *Polydactylus siamensis* Motomura, Iwatsuki & Yoshino 2001.

Etymology: the generic name refers to the thread-like fin rays of the pectoral fin and the diagnostic stripes along the lateral flanks [*fili* (Latin) = thread and *striatus* (Latin) = striped]. Gender masculine.

Remarks: “*Polydactylus*” *siamensis* was characterized by several longitudinal dark stripes along the flank, 5 pectoral filaments, and 54–58 lateral-line scales (Motomura, Iwatsuki & Yoshino, 2001). Although we could not sample this taxon in this study, the species has the diagnostic characters of *Filistriatus*. The species is also reassigned to the new genus.

### Reassessment of *Leptomelanosoma* Motomura & Iwatsuki 2001

Diagnosis: a genus of the Polynemidae with the following combination of characters: ethmoid not covered dorsally by frontals, basisphenoid posteriorly displaced, prootic excluded from rear margin of orbit by pronounced expansion of pterosphenoid.

Type species: *Polydactylus indicus* (Shaw 1804)

Included species: *Polydactylus indicus* (Shaw 1804), *Polydactylus macrophthalmus* (Bleeker 1858)

Remarks: although not included in the description by Motomura & Iwatsuki (2001a), the lateral-line scales in specimens of *L. indicum* are poorly pigmented and nearly white along the length of the flank in both fresh and preserved specimens. Further, the scales immediately above and below the lateral line are densely pigmented, accentuating the lateral-line scales in this taxon. In specimens of *L. macrophthalmus*, a similar condition exists in the lateral line, particularly in the scales anterior to the anal fin of preserved specimens (see Motomura et al, 2001, fig. 2). We did not include this lateral-line pigmentation as diagnostic for the genus as we could not locate images of freshly caught specimens of *L. macrophthalmus*. Subsequent works should explore this character as a diagnostic feature of the genus.

## Discussion

### Species and relationships of *Filistriatus*

*Filistriatus bifurcus* is a medium-sized threadfin (119–272 mm SL; Motomura, 2004) currently only known from shallow waters along the southern coast of Indonesia and the southwestern coast of Taiwan. The species was described from a single specimen captured near Lombok Island, Indonesia, based on differences in fin-ray and lateral-line scale counts, robustness of the second dorsal-fin spine, and bifurcation of the lateral line on the caudal fin. The bifurcation of the lateral line on the caudal fin has been documented in a few species of threadfins, including two species of *Eleutheronema* and species of *Polydactylus* that occur in the New World (Motomura, 2004). Some species of *Eleutheronema* have two bifurcations of the lateral line on the caudal fin, with a secondary bifurcation occurring on the lower lobe (Motomura, 2004). *Filistriatus bifurcus* was further characterized by 8–9 dark stripes above the lateral line and 8–9 faint stripes below the lateral line, along longitudinal flank scale rows. Only three species of threadfin are known to have similar striped patterns above and below the lateral line: *F. plebeius*, *F. siamensis*, and *F. sexfilis*. These three species are largely sympatric over their respective ranges (Feltes, 1986; Motomura, Iwatsuki & Yoshino, 2001; Motomura, Iwatsuki & Kimura, 2001; Motomura, 2004), with *F. plebeius* occurring as far west as the eastern coast of Africa, *F. sexfilis* occurring as far east as the Hawaiian Islands, and *F. siamensis* found only in the waters around Thailand. Feltes (1986: 516) notes that *F. plebeius* and *F. sexfilis* are “so closely related” that differentiating individual specimens can be difficult. *Filistriatus plebeius* and *F. sexfilis* have been included in three analyses on polynemid intrarelationships, being recovered either as a clade (Kang, 2017; Girard et al., 2022a) or as close allies in a clade with *Eleutheronema* and “*P.*” *opercularis* (Presti, Johnson & Datovo, 2023)*.* However, *F. bifurcus* and *F. siamensis* have yet to be included in an analysis.

**Figure 5:**
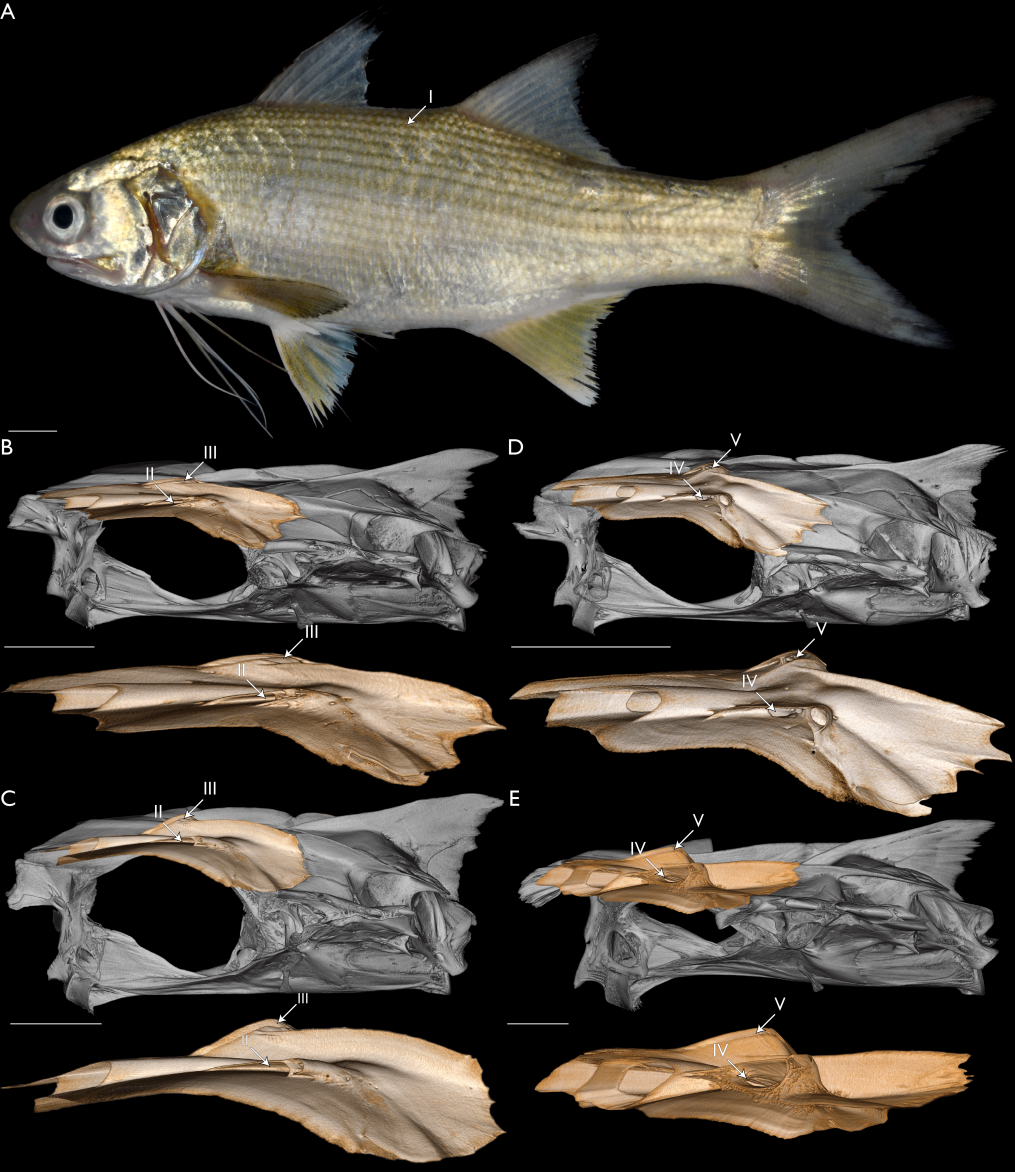
Morphology relating to the monophyly of * Filistriatus* gen. nov. (A) Fresh specimen of *F. plebeius* gen. nov. (USNM 471329) in lateral view. (B) Neurocranium of *F. bifurcus* gen. nov. (USNM 76627) in dorsolateral view. (C) Neurocranium of *F. sexfilis* gen. nov. (CSIRO C261) in dorsolateral view. (D) Neurocranium of *P. virginicus* (FMNH 104648) in dorsolateral view. (E) Neurocranium of *L. indicum* (USNM 357716) in dorsolateral view. Left frontal and medial supraorbital canal commissure colored in (B–E), with isolated colored frontals below each whole neurocranium. Arrow I indicates dark stripes along the longitudinal scale rows above the lateral line. Faint stripes also present along the longitudinal scale rows below the lateral line. Arrow II indicates reduced lateral pore of the supraorbital canal. Arrow III indicates reduced medial commissure of sensory canal on the dorsal surface of the frontal. Arrow IV indicates pronounced lateral pore of the supraorbital canal that is visible when viewing the side of the neurocranium. Arrow V indicates elevated medial commissure of sensory canal. Scale bars = five mm.

The mitogenome of *F. bifurcus* was obtained through the extraction of hDNA from the liver of a 100+-year-old museum specimen. The combined analysis of these data with mitochondrial and UCE datasets recovered *F. bifurcus* in a clade with *F. plebeius* and *F. sexfilis*. Several counts and measurements among species of *Filistriatus* support the recovered relationship, including similar body depth at first dorsal-fin origin relative to SL (*F. bifurcus*: 26–28%; *F. plebeius*: 25–34%; *F. sexfilis*: 27–34%), upper-jaw length relative to SL (*F. bifurcus*: 14%; *F. plebeius* and *F. sexfilis*: 13–16% for both), and number of pored lateral-line scales (*F. bifurcus*: 69–72; *F. plebeius*: 60–68; *F. sexfilis*: 60–67). Further, the presence of 7–9 dark longitudinal stripes above and below the lateral line (see above; Motomura, Kimura & Iwatsuki, 2001; Motomura, 2004) is unique among members of the family. Characters in the neurocranium also support these taxa being closely related, including a reduced lateral pore of the supraorbital canal and reduced canal commissure on the dorsal surface of the frontal. In their assessment of morphological variation across species of threadfins that occur near Bombay, Marathe & Bal (1958) noted morphological variation in the supraorbital sensory canal in all species of threadfins they examined. Typically, this canal has a pronounced lateral pore that is visible when viewing the lateral aspect of the neurocranium and a medial commissure that is elevated and open mesially. In species of *Filistriatus*, the lateral pore is reduced, not visible within the lateral triangular aperture of the frontal (Fig. 5). Additionally, the canal commissure is reduced, with a distinct lowering of the dorsal surface compared to other species of *Polydactylus* (Fig. 5). Based on these morphological features, overlapping meristics, and unique pigmentation pattern among threadfins, we find strong support for a clade of *Filistriatus*, as recovered in the analysis of mitochondrial and UCE loci.

### Species and relationships of *Leptomelanosoma*

*Leptomelanosoma indicum* is a widely distributed species of threadfin, found from Pakistan to Papua New Guinea (Motomura, 2004). The species was described as *Polynemus indicus* Shaw 1804 based on an illustrated specimen from India (see Russell, 1803: 68, fig. 184) and later attributed to the genus *Polydactylus* by Myers (1936). Marathe & Bal (1958) noted several characters that are unique to the species among threadfins, including a large ethmoid extending anteriorly and exposed dorsally by the frontals, an oval-shaped vomerine tooth plate, a posteriorly displaced basisphenoid, a dorsally exposed sphenotic, and an enlarged palatine. Motomura & Iwatsuki (2001a) described a new genus, *Leptomelanosoma*, for the taxon based on the poorly developed lip adjacent to the lower jaw, the gas bladder with numerous lateral appendages, the ethmoid exposed dorsally by frontals, the anterior one-third of lower jaw with small teeth extending onto the lateral surface, the sphenotics exposed on the margin of neurocranium, filamentous caudal-fin rays, the vomer with oval-shaped tooth plate, and the wide tooth plates on palatine and ectopterygoid. While diagnostic to *Leptomelanosoma*, the poorly developed lower-jaw lip and filamentous caudal-fin rays were listed as homoplastic with the monospecific genus *Parapolynemus* by Motomura & Iwatsuki (2001a), and several other characters, such as the gas bladder with lateral appendages and sphenotics visible on the dorsal margin of the neurocranium, were found to be autapomorphic among threadfins (Motomura & Iwatsuki, 2001a). Presti, Johnson & Datovo (2023) recovered *L. indicum* sister to a clade of *Parapolynemus* and *Polynemus* based on nine morphological characters. They also identified 14 autapomorphic characters for *Leptomelanosoma*, including articulation between the metapterygoid and endopterygoid (their character 38) and fusion of the endopterygoid and ectopterygoid (their character 48). The study by Girard et al. (2022a) recovered *L. indicum* sister to a clade of *Filimanus* and the black-spotted species of “*Polydactylus*” but did not report any morphological characters that supported this hypothesis.

*Leptomelanosoma macrophthalmus* (Bleeker 1858) was originally described in the genus *Polynemus* based on two specimens from Indonesia. Only known to occur in rivers on two Indonesian islands, the Kapuas River in Kalimantan and the Batanghari and Musi Rivers in Sumatra, this species is thought to have the most-restricted distribution of any species within the family (Motomura, Iwatsuki & Kimura, 2001). The taxon was considered a species of *Polynemus* until Feltes (1993) included it in *Polydactylus.* Motomura, Iwatsuki & Kimura (2001) redescribed the species, diagnosing it by the presence of villiform teeth in broad bands on the vomer, palatine, and ectopterygoid, elongate pectoral-fin rays, and a well-developed gas bladder, among other characters. No previous studies have included this taxon in a phylogenetic analysis.

**Figure 6:**
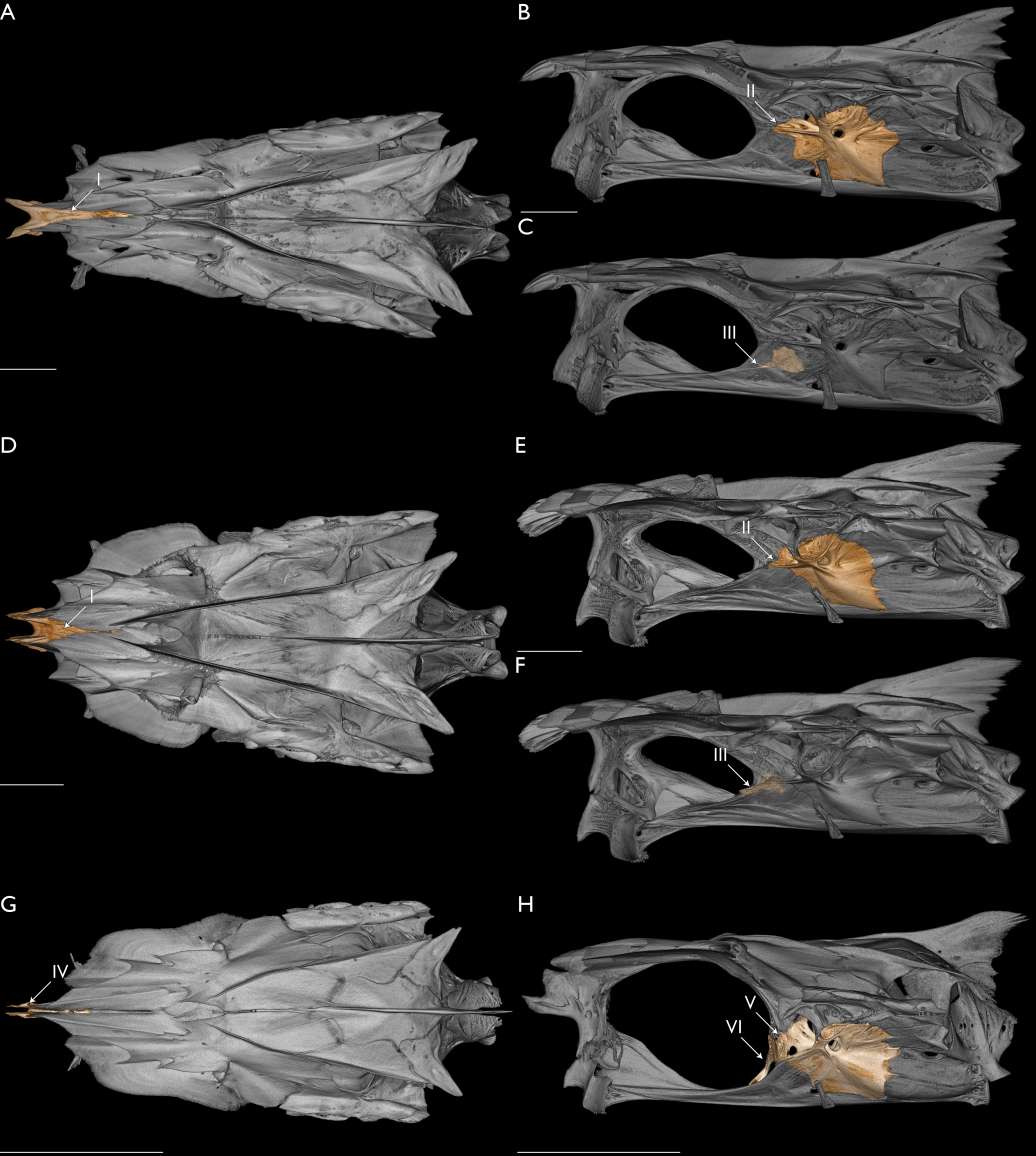
Morphology relating to the monophyly of *Leptomelanosoma*. Neurocranium of *L. macrophthalmus* (UMMZ 171714) in dorsal (A) and lateral (B and C) views. Neurocranium of *L. indicum* (USNM 357716) in dorsal (D) and lateral (E and F) views. Neurocranium of *Polydactylus virginicus* (FMNH 104648) in dorsal (G) and lateral (H) views. (A, D, G) Ethmoid colored. (B, E, H) Prootic colored. (C, F, H) Basisphenoid colored. Opacity of neurocranium reduced in (C) and (F) so basisphenoid position could be visualized. Arrow I indicates ethmoid not covered by frontals and exposed dorsally. Arrow II indicates prootic excluded from rear margin of orbit by pronounced expansion of pterosphenoid. Arrow III indicates basisphenoid posteriorly displaced. Arrow IV indicates ethmoid covered by frontals. Arrow V indicates prootic included in rear margin of orbit, separating parasphenoid from pterosphenoid. Arrow VI indicates basisphenoid largely vertical and anterior to the posterior margin of the orbit. Scale bars = five mm.

The mitochondrial genome of *L. macrophthalmus* was acquired through the extraction of hDNA from the liver of a 20+-year-old museum specimen. The combined analyses of these data with mitochondrial and UCE loci recovered *L. macrophthalmus* sister to *L. indicum*. When examining the morphology of *L. macrophthalmus*, we find four characters in the neurocranium that support a sister-group relationship between these taxa, including characters once considered to be unique to *L. indicum* (Fig. 6). The typical condition in polynemid neurocrania is for the anterior margin of the frontal to extend rostrally above the ethmoid and to meet at a discrete point (see Marathe & Bal, 1958, fig. 2; Motomura & Iwatsuki, 2001a, fig. 4). One of the diagnostic characters of *Leptomelanosoma* is the truncation of the frontal and separation from the opposing frontal rostrally, such that the dorsal margin of the ethmoid is exposed (Marathe & Bal, 1958; Motomura & Iwatsuki, 2001a; Fig. 6). When examining the neurocranium of *L. macrophthalmus*, the anterior margin of the frontal is also truncated and distinctly separated from the opposing frontal, with the dorsal margin of the ethmoid visible (Fig. 6). This character has not been previously mentioned for this or any other species of polynemid outside of *L. indicum* (Motomura, Iwatsuki & Kimura, 2001; Motomura, 2004). Across polynemid genera, several character states are found within the posterior margin of the orbit, the position of the basisphenoid, the shape of the prootic, and the interaction between the parasphenoid, prootic, and pterosphenoid. The typical condition for species of *Polydactylus* is for the basisphenoid to be largely vertical and positioned anterior to the posterior margin of the orbit. Further, the prootic typically contributes to the posterior margin of the orbit, separating the parasphenoid from the pterosphenoid. Marathe & Bal (1958) noted that the basisphenoid is not visible in lateral view in *L. indicum* and we find a similar condition in *L. macrophthalmus* (Fig. 6). The basisphenoid in both taxa is posteriorly displaced and reclined within the cranial vault, but remains in contact with the pterosphenoid and prootic, as is typical in polynemids broadly (Feltes, 1986; Feltes, 1991; Feltes, 1993). Presti, Johnson & Datovo (2023) found that the prootic was excluded from the posterior margin of the orbit in *L. indicum* as well as *Eleutheronema*, *Parapolynemus*, *Pentanemus*, and *Polynemus*. Feltes (1993) noted that the condition in *Eleutheronema* was different than that of *Parapolynemus* and *Polynemus*, as exclusion of the prootic in *Eleutheronema* was by a slight connection between the anteroventral corner of the pterosphenoid and anterodorsal corner of the parasphenoid *versus* the extensive expansion of the pterosphenoid, dorsal extension of the parasphenoid, and broad point of contact between the parasphenoid and pterosphenoid in *Parapolynemus* and *Polynemus*. In the specimens we examined, we did not find the prootic excluded from the posterior margin of the orbit in *Pentanemus*. The contact between the pterosphenoid and parasphenoid is broader in *Leptomelanosoma* than in *Eleutheronema*, with the pterosphenoid having a substantial anteroventral arm that extends to and interacts with the parasphenoid. The lateral arm of the prootic that reaches towards the sphenotic is also shortened, not extending to the posterior margin of the orbit. The contact between the parasphenoid and pterosphenoid in *Leptomelanosoma* is not as broad as what is seen in *Parapolynemus* and *Polynemus*, where the pterosphenoid is extensively expanded (Feltes, 1993). Based on these morphological features, we find strong support for the inclusion of *L. macrophthalmus* in the genus *Leptomelanosoma*, as recovered with molecular data.

## Conclusions

### Historical DNA can reliably inform modern phylogenetic analyses

This study builds on the recent success of sequencing DNA from historic and/or formalin-fixed specimens, successfully obtaining sequence data from fluid-preserved museum specimens using a commercial-kit-based approach. The resulting mitochondrial data from these specimens allowed for two enigmatic taxa, *F. bifurcus* and *L. macrophthalmus*, to be placed in an evolutionary context for the first time. This new phylogenetic framework allowed for the further study of internal morphology and subsequent modifications to the classification of “*Polydactylus*” based on both genomic and anatomical characters, including the description of a new genus. This result further supports analyzing historic samples alongside a robust dataset of molecular markers generated from modern samples can inform modern phylogenetic analyses. The methods described here add to the growing body of literature of methods supporting the utility of hDNA to genetically characterize type specimens, to aid in accounting for genetic diversity within species complexes, and to improve our understanding of taxonomy and classification across groups of animals that have millions of historically inaccessible specimens for genomic approaches.

### Collections concerns surrounding the genomic potential of museum specimens

Targets for destructive hDNA sampling typically represent rare, extinct, taxonomically, and historically significant specimens (*e.g.*, Palandačić et al., 2024; Muschick, Rüber & Matschiner, 2025). As museum specimens are irreplaceable records of biodiversity, researchers and collection staff should work collaboratively to balance the growing demands for destructive genomic research with the long-term conservation of specimens for other research needs. We performed minimally invasive dissections to extract liver samples through small abdominal incisions. Prioritizing both specimen integrity and genetic sampling allowed for genomic and morphological approaches to be applied, classification and taxonomy to be revised, and our understanding of threadfin evolution to be improved. While the techniques presented in this study can be applied to specimens greater than 50 mm SL, successfully sampling hDNA from smaller specimens and maintaining an intact morphological vouchers presents additional challenges (*e.g.*, Muschick, Rüber & Matschiner, 2025). As technology and methods continue to improve, the likelihood of acquiring viable hDNA from muscle punches or external features will increase, decreasing the need for invasive sampling from internal organs. Until those methods are developed, it is critical that both answering new questions and specimen preservation are balanced, ensuring these unique records are available to future researchers.

##  Supplemental Information

10.7717/peerj.20029/supp-1 Supplemental Information 1 Museum catalog and GenBank information

10.7717/peerj.20029/supp-2 Supplemental Information 2 Log file for partitioning schemen and model analysis

10.7717/peerj.20029/supp-3 Supplemental Information 3 Partitions and associated models

10.7717/peerj.20029/supp-4 Supplemental Information 4 Log file for phylogenetic analysis

10.7717/peerj.20029/supp-5 Supplemental Information 5 Tre file of Fig. 3

10.7717/peerj.20029/supp-6 Supplemental Information 6 MapDamage2 output from analyses of hDNA samples

## Additional Information and Declarations

### DNA Deposition

The following information was supplied regarding the deposition of DNA sequences: The annotated mitochondrial genomes are available at GenBank: PV590073 –PV590096; PV590343 –PV590344; PV593525; SRR33326234 –SRR33326248.

### Data Availability

The following information was supplied regarding data availability: The annotated mitochondrial genomes are available at GenBank: PV590073 –PV590096; PV590343 –PV590344; PV593525; SRR33326234 –SRR33326248. The models are available at MorphoSource: https://www.morphosource.org/projects/000735880. Users are required to request a loan of the scans either by contacting the museums or using the MorphoSource “Request Media” or “Requests Download” functionality: – https://doi.org/10.17602/M2/M735960: Diane Pitassy, pitassyd@si.edu ; – https://doi.org/10.17602/M2/M735955: Diane Pitassy, pitassyd@si.edu ; – https://doi.org/10.17602/M2/M735950: Diane Pitassy, pitassyd@si.edu ; – https://doi.org/10.17602/M2/M735945: Helen O’Niell, helen.oneill@csiro.au ; Nicole Fisher, nicole.fisher@csiro.au ; – https://doi.org/10.17602/M2/M735940: Caleb McMahan, cmcmahan@fieldmuseum.org ; – https://doi.org/10.17602/M2/M735937: Hernan Lopez-Fernandez, hlopezf@umich.edu . Phylogenetic analyses were performed on the High Performance Cluster (SI/HPC), Smithsonian Institution https://doi.org/10.25572/SIHPC.

### New Species Registration

The following information was supplied regarding the registration of a newly described species: Publication LSID: urn:lsid:zoobank.org:pub:E0E70F44-3417-4BD9-87EF-F4F2CD157F68. *Filistriatus* Genus LSID: urn:lsid:zoobank.org:act:983362F8-2F4E-4730-A9BE-BE24F2784474.
