# Supplementary material for: Unlocking the genomic potential of historical and formalin-fixed specimens: phylogenetic insights from museum-preserved threadfin fishes (Teleostei: Polynemidae)
Source: PeerJ. 2025 Sep 30;13:e20029. doi: 10.7717/peerj.20029 (PMC13353053; doi:10.7717/peerj.20029)
Supplement: Supplemental Information 3 [file peerj-13-20029-s003.pdf]

Partiton Subset	Best Fitting Model	Region(s) in Partition
UCE Partition 1	GTR+G	UCE 1001 Left Region
UCE Partition 2	GTR+G	UCE 693 Core Region, UCE 1001 Core Region, UCE 444 Core Region, UCE 781 Core Region
UCE Partition 3	GTR+G	UCE 46 Left Region, UCE 1001 Right Region, UCE 143 Left Region, UCE 695 Right Region
UCE Partition 4	GTR+G	UCE 520 Core Region, UCE 1007 Left Region, UCE 288 Right Region
UCE Partition 5	GTR+I+G	UCE 1007 Core Region, UCE 561 Left Region, UCE 649 Core Region, UCE 769 Core Region
UCE Partition 6	GTR+G	UCE 1007 Right Region
UCE Partition 7	GTR+I+G	UCE 191 Core Region, UCE 1224 Core Region, UCE 556 Core Region, UCE 1017 Left Region, UCE 402 Left Region, UCE 609 Left Region, UCE 21 Left Region
UCE Partition 8	GTR+G	UCE 1017 Core Region, UCE 723 Left Region, UCE 444 Right Region, UCE 565 Right Region
UCE Partition 9	GTR+I+G	UCE 1017 Right Region, UCE 408 Left Region, UCE 706 Right Region, UCE 267 Left Region
UCE Partition 10	GTR+G	UCE 1018 Left Region, UCE 981 Left Region
UCE Partition 11	GTR+I+G	UCE 282 Left Region, UCE 149 Core Region, UCE 1018 Core Region
UCE Partition 12	GTR+I+G	UCE 180 Left Region, UCE 1018 Right Region
UCE Partition 13	GTR+G	UCE 376 Left Region, UCE 1020 Left Region
UCE Partition 14	GTR+G	UCE 192 Left Region, UCE 1020 Core Region
UCE Partition 15	GTR+I+G	UCE 196 Core Region, UCE 1020 Right Region
UCE Partition 16	GTR+G	UCE 1027 Left Region, UCE 992 Right Region
UCE Partition 17	GTR+I+G	UCE 358 Core Region, UCE 176 Core Region, UCE 129 Core Region, UCE 1027 Core Region, UCE 460 Right Region
UCE Partition 18	GTR+G	UCE 1027 Right Region, UCE 736 Left Region, UCE 176 Left Region
UCE Partition 19	GTR+I+G	UCE 181 Right Region, UCE 103 Left Region
UCE Partition 20	GTR+G	UCE 47 Right Region, UCE 185 Right Region, UCE 103 Core Region
UCE Partition 21	GTR+G	UCE 214 Left Region, UCE 103 Right Region
UCE Partition 22	GTR+G	UCE 885 Left Region, UCE 471 Left Region, UCE 503 Left Region, UCE 358 Left Region, UCE 1032 Left Region, UCE 446 Left Region
UCE Partition 23	GTR+I+G	UCE 503 Core Region, UCE 175 Right Region, UCE 1032 Core Region, UCE 416 Core Region
UCE Partition 24	GTR+G	UCE 1032 Right Region
UCE Partition 25	GTR+I+G	UCE 1036 Left Region, UCE 535 Left Region, UCE 509 Right Region, UCE 1036 Right Region, UCE 1169 Right Region, UCE 509 Core Region
UCE Partition 26	GTR+I+G	UCE 1036 Core Region
UCE Partition 27	GTR+G	UCE 168 Right Region, UCE 647 Right Region, UCE 1038 Left Region, UCE 553 Right Region
UCE Partition 28	GTR+I+G	UCE 1038 Core Region, UCE 1105 Core Region
UCE Partition 29	GTR+G	UCE 1038 Right Region
UCE Partition 30	GTR	UCE 1040 Left Region, UCE 446 Right Region
UCE Partition 31	GTR+G	UCE 252 Core Region, UCE 1040 Core Region, UCE 166 Core Region, UCE 183 Core Region
UCE Partition 32	GTR+G	UCE 643 Left Region, UCE 1040 Right Region
UCE Partition 33	GTR+G	UCE 1251 Left Region, UCE 1279 Right Region, UCE 1042 Left Region, UCE 149 Left Region, UCE 934 Right Region, UCE 1048 Left Region, UCE 199 Left Region, UCE 535 Core Region, UCE 167 Left Region
UCE Partition 34	GTR+I+G	UCE 815 Left Region, UCE 1338 Left Region, UCE 1042 Core Region, UCE 1293 Core Region
UCE Partition 35	GTR+I+G	UCE 243 Left Region, UCE 1106 Core Region, UCE 364 Left Region, UCE 1287 Right Region, UCE 335 Right Region, UCE 1042 Right Region, UCE 699 Left Region
UCE Partition 36	GTR+G	UCE 569 Core Region, UCE 1044 Left Region, UCE 990 Left Region, UCE 467 Left Region, UCE 769 Left Region
UCE Partition 37	GTR+I+G	UCE 1044 Core Region, UCE 51 Right Region
UCE Partition 38	GTR+G	UCE 1044 Right Region
UCE Partition 39	GTR+G	UCE 120 Right Region, UCE 556 Right Region, UCE 159 Right Region, UCE 1046 Left Region, UCE 532 Left Region, UCE 852 Core Region
UCE Partition 40	GTR+G	UCE 1046 Core Region
UCE Partition 41	GTR+I+G	UCE 622 Core Region, UCE 1046 Right Region, UCE 642 Right Region, UCE 1191 Right Region, UCE 203 Left Region
UCE Partition 42	GTR+I+G	UCE 1048 Core Region, UCE 353 Right Region
UCE Partition 43	GTR+G	UCE 1048 Right Region
UCE Partition 44	GTR+G	UCE 321 Core Region, UCE 157 Core Region, UCE 944 Core Region, UCE 861 Core Region, UCE 748 Core Region, UCE 757 Left Region, UCE 1053 Left Region
UCE Partition 45	GTR+I+G	UCE 159 Core Region, UCE 1279 Core Region, UCE 527 Right Region, UCE 526 Core Region, UCE 1273 Core Region, UCE 1061 Core Region, UCE 1113 Core Region, UCE 1053 Core Region
UCE Partition 46	GTR+G	UCE 1175 Left Region, UCE 152 Left Region, UCE 1053 Right Region
UCE Partition 47	GTR+I+G	UCE 1054 Left Region, UCE 640 Core Region, UCE 363 Right Region
UCE Partition 48	GTR	UCE 1054 Core Region
UCE Partition 49	GTR+G	UCE 653 Right Region, UCE 860 Left Region, UCE 1054 Right Region, UCE 191 Left Region, UCE 837 Left Region, UCE 1279 Left Region
UCE Partition 50	GTR+I+G	UCE 1056 Left Region
UCE Partition 51	GTR+G	UCE 1056 Core Region
UCE Partition 52	GTR+I+G	UCE 495 Right Region, UCE 458 Core Region, UCE 1056 Right Region
UCE Partition 53	GTR+G	UCE 572 Left Region, UCE 547 Right Region, UCE 410 Left Region, UCE 1061 Right Region, UCE 106 Left Region, UCE 713 Left Region, UCE 420 Left Region
UCE Partition 54	GTR+I+G	UCE 106 Core Region, UCE 411 Core Region, UCE 151 Core Region, UCE 307 Core Region
UCE Partition 55	GTR+G	UCE 106 Right Region
UCE Partition 56	GTR+G	UCE 1204 Right Region, UCE 383 Left Region, UCE 1061 Left Region, UCE 120 Left Region, UCE 274 Left Region
UCE Partition 57	GTR+I+G	UCE 1062 Left Region, UCE 161 Core Region, UCE 57 Core Region, UCE 570 Core Region
UCE Partition 58	GTR+G	UCE 461 Left Region, UCE 1062 Core Region
UCE Partition 59	GTR+G	UCE 372 Right Region, UCE 162 Right Region, UCE 1062 Right Region
UCE Partition 60	GTR+I+G	UCE 174 Core Region, UCE 768 Left Region, UCE 1064 Left Region
UCE Partition 61	GTR+G	UCE 1064 Core Region
UCE Partition 62	GTR+I+G	UCE 1228 Core Region, UCE 1064 Right Region, UCE 966 Left Region
UCE Partition 63	GTR+G	UCE 1069 Left Region, UCE 833 Core Region
UCE Partition 64	GTR+I+G	UCE 1269 Core Region, UCE 749 Right Region, UCE 535 Right Region, UCE 1069 Core Region, UCE 54 Right Region
UCE Partition 65	GTR+I+G	UCE 1069 Right Region, UCE 320 Left Region
UCE Partition 66	GTR+I+G	UCE 1075 Left Region
UCE Partition 67	GTR+G	UCE 1075 Core Region
UCE Partition 68	GTR+G	UCE 1075 Right Region
UCE Partition 69	GTR+G	UCE 1273 Left Region, UCE 108 Left Region, UCE 737 Left Region
UCE Partition 70	GTR+I+G	UCE 653 Core Region, UCE 808 Core Region, UCE 108 Core Region
UCE Partition 71	GTR+G	UCE 309 Left Region, UCE 934 Left Region, UCE 363 Left Region, UCE 685 Left Region, UCE 108 Right Region, UCE 982 Left Region, UCE 310 Left Region, UCE 175 Left Region, UCE 705 Left Region
UCE Partition 72	GTR+G	UCE 769 Right Region, UCE 124 Left Region, UCE 327 Left Region, UCE 1080 Left Region, UCE 302 Core Region
UCE Partition 73	GTR+G	UCE 418 Core Region, UCE 1080 Core Region
UCE Partition 74	GTR+I+G	UCE 1184 Core Region, UCE 737 Core Region, UCE 1080 Right Region, UCE 363 Core Region, UCE 915 Core Region
UCE Partition 75	GTR+G	UCE 183 Right Region, UCE 885 Core Region, UCE 575 Left Region, UCE 600 Core Region, UCE 1087 Left Region, UCE 851 Right Region
UCE Partition 76	GTR+G	UCE 997 Core Region, UCE 372 Core Region, UCE 1087 Core Region, UCE 136 Left Region
UCE Partition 77	GTR+G	UCE 1087 Right Region, UCE 431 Right Region
UCE Partition 78	GTR+G	UCE 109 Left Region, UCE 975 Core Region
UCE Partition 79	GTR+I+G	UCE 109 Core Region, UCE 1295 Core Region, UCE 824 Core Region, UCE 214 Core Region
UCE Partition 80	GTR+I+G	UCE 109 Right Region
UCE Partition 81	GTR+I+G	UCE 327 Core Region, UCE 697 Core Region, UCE 1093 Left Region, UCE 399 Left Region
UCE Partition 82	GTR+G	UCE 993 Left Region, UCE 1093 Core Region, UCE 3 Core Region
UCE Partition 83	GTR+G	UCE 1242 Left Region, UCE 1277 Core Region, UCE 468 Core Region, UCE 1093 Right Region, UCE 546 Core Region

Partiton Subset	Best Fitting Model	Region(s) in Partition
UCE Partition 84	GTR+G	UCE 1097 Left Region
UCE Partition 85	GTR+I+G	UCE 624 Core Region, UCE 47 Core Region, UCE 31 Core Region, UCE 1097 Core Region, UCE 418 Left Region
UCE Partition 86	GTR+G	UCE 1097 Right Region
UCE Partition 87	GTR+G	UCE 1105 Left Region
UCE Partition 88	GTR+G	UCE 1105 Right Region, UCE 819 Left Region, UCE 162 Left Region
UCE Partition 89	GTR+G	UCE 1106 Left Region, UCE 1160 Right Region, UCE 908 Right Region, UCE 283 Core Region
UCE Partition 90	GTR+G	UCE 139 Right Region, UCE 1106 Right Region, UCE 870 Left Region, UCE 547 Core Region, UCE 553 Core Region
UCE Partition 91	GTR+I+G	UCE 1108 Left Region
UCE Partition 92	GTR+G	UCE 706 Core Region, UCE 1108 Core Region
UCE Partition 93	GTR+G	UCE 1108 Right Region, UCE 813 Left Region, UCE 1234 Right Region, UCE 811 Right Region, UCE 669 Left Region, UCE 1234 Left Region
UCE Partition 94	GTR+G	UCE 510 Left Region, UCE 1110 Left Region
UCE Partition 95	GTR+I+G	UCE 660 Core Region, UCE 118 Core Region, UCE 1110 Core Region, UCE 1296 Core Region
UCE Partition 96	GTR+G	UCE 1256 Right Region, UCE 124 Right Region, UCE 1110 Right Region
UCE Partition 97	GTR+G	UCE 1111 Left Region
UCE Partition 98	GTR	UCE 1111 Core Region
UCE Partition 99	GTR+G	UCE 1111 Right Region
UCE Partition 100	GTR+I+G	UCE 1113 Left Region, UCE 1133 Core Region, UCE 713 Core Region
UCE Partition 101	GTR+G	UCE 1113 Right Region, UCE 89 Right Region
UCE Partition 102	GTR+G	UCE 504 Right Region, UCE 1119 Left Region
UCE Partition 103	GTR+G	UCE 1119 Core Region, UCE 777 Left Region
UCE Partition 104	GTR+G	UCE 1119 Right Region, UCE 709 Right Region, UCE 113 Left Region, UCE 372 Left Region
UCE Partition 105	GTR+I+G	UCE 113 Core Region, UCE 238 Left Region, UCE 574 Right Region, UCE 565 Core Region
UCE Partition 106	GTR+G	UCE 113 Right Region, UCE 282 Core Region
UCE Partition 107	GTR+G	UCE 1133 Left Region
UCE Partition 108	GTR+G	UCE 569 Left Region, UCE 1133 Right Region, UCE 663 Left Region, UCE 982 Right Region, UCE 1270 Right Region
UCE Partition 109	GTR+G	UCE 344 Core Region, UCE 879 Left Region, UCE 1139 Left Region, UCE 359 Core Region
UCE Partition 110	GTR+I+G	UCE 692 Left Region, UCE 494 Core Region, UCE 321 Right Region, UCE 73 Core Region, UCE 495 Left Region, UCE 1139 Core Region, UCE 38 Core Region
UCE Partition 111	GTR+G	UCE 526 Right Region, UCE 648 Core Region, UCE 731 Right Region, UCE 1139 Right Region, UCE 1200 Core Region, UCE 3 Left Region, UCE 30 Left Region
UCE Partition 112	GTR+I+G	UCE 1143 Left Region, UCE 181 Left Region, UCE 1143 Right Region
UCE Partition 113	GTR+I+G	UCE 1169 Left Region, UCE 1143 Core Region, UCE 598 Right Region
UCE Partition 114	GTR+G	UCE 1295 Right Region, UCE 536 Left Region, UCE 115 Left Region, UCE 170 Left Region
UCE Partition 115	GTR+G	UCE 115 Core Region
UCE Partition 116	GTR+G	UCE 115 Right Region, UCE 427 Right Region, UCE 605 Left Region, UCE 932 Right Region, UCE 1238 Core Region, UCE 651 Left Region, UCE 968 Right Region
UCE Partition 117	GTR+G	UCE 1154 Left Region
UCE Partition 118	GTR+G	UCE 64 Core Region, UCE 802 Right Region, UCE 143 Core Region, UCE 1154 Core Region
UCE Partition 119	GTR+G	UCE 1263 Left Region, UCE 697 Left Region, UCE 1253 Core Region, UCE 1154 Right Region, UCE 568 Core Region, UCE 762 Left Region, UCE 99 Right Region, UCE 1277 Right Region, UCE 588 Right Region, UCE 391 Core Region, UCE 364 Core Region
UCE Partition 120	GTR+G	UCE 713 Right Region, UCE 116 Left Region, UCE 335 Left Region, UCE 761 Left Region
UCE Partition 121	GTR+G	UCE 116 Core Region
UCE Partition 122	GTR+G	UCE 116 Right Region
UCE Partition 123	GTR+G	UCE 494 Left Region, UCE 1160 Left Region, UCE 685 Right Region, UCE 394 Right Region, UCE 30 Right Region, UCE 73 Left Region
UCE Partition 124	GTR+G	UCE 1160 Core Region, UCE 467 Core Region
UCE Partition 125	GTR+G	UCE 67 Right Region, UCE 1168 Left Region
UCE Partition 126	GTR+G	UCE 1168 Core Region, UCE 852 Right Region
UCE Partition 127	GTR+G	UCE 494 Right Region, UCE 672 Right Region, UCE 76 Left Region, UCE 532 Right Region, UCE 1168 Right Region
UCE Partition 128	GTR+I+G	UCE 1169 Core Region, UCE 731 Left Region
UCE Partition 129	GTR+I+G	UCE 756 Right Region, UCE 1173 Left Region, UCE 295 Core Region
UCE Partition 130	GTR+I+G	UCE 819 Core Region, UCE 1192 Core Region, UCE 67 Core Region, UCE 934 Core Region, UCE 187 Core Region, UCE 1173 Core Region, UCE 1282 Left Region, UCE 60 Core Region
UCE Partition 131	GTR+G	UCE 1173 Right Region, UCE 948 Left Region, UCE 353 Left Region
UCE Partition 132	GTR+G	UCE 795 Core Region, UCE 351 Core Region, UCE 1175 Core Region, UCE 288 Core Region
UCE Partition 133	GTR+G	UCE 187 Right Region, UCE 199 Right Region, UCE 1175 Right Region, UCE 575 Core Region, UCE 172 Right Region
UCE Partition 134	GTR+G	UCE 276 Core Region, UCE 247 Left Region, UCE 1286 Left Region, UCE 220 Core Region, UCE 837 Right Region, UCE 118 Left Region, UCE 38 Right Region, UCE 761 Right Region
UCE Partition 135	GTR+G	UCE 31 Left Region, UCE 118 Right Region, UCE 666 Right Region
UCE Partition 136	GTR+I+G	UCE 1184 Left Region, UCE 89 Left Region, UCE 723 Core Region, UCE 966 Right Region
UCE Partition 137	GTR+G	UCE 411 Right Region, UCE 1184 Right Region
UCE Partition 138	GTR+G	UCE 885 Right Region, UCE 1186 Left Region, UCE 1276 Right Region, UCE 496 Left Region, UCE 392 Right Region
UCE Partition 139	GTR+I+G	UCE 414 Core Region, UCE 690 Core Region, UCE 1186 Core Region
UCE Partition 140	GTR+G	UCE 1186 Right Region, UCE 368 Left Region
UCE Partition 141	GTR+I+G	UCE 34 Core Region, UCE 1191 Left Region, UCE 952 Core Region, UCE 274 Core Region, UCE 170 Right Region, UCE 663 Core Region
UCE Partition 142	GTR+G	UCE 348 Right Region, UCE 1191 Core Region
UCE Partition 143	GTR+G	UCE 832 Right Region, UCE 997 Right Region, UCE 1192 Left Region
UCE Partition 144	GTR+G	UCE 1192 Right Region
UCE Partition 145	GTR+I+G	UCE 1263 Core Region, UCE 120 Core Region, UCE 577 Core Region, UCE 368 Core Region
UCE Partition 146	GTR+G	UCE 427 Left Region, UCE 1200 Left Region, UCE 547 Left Region, UCE 157 Right Region, UCE 718 Right Region, UCE 1277 Left Region
UCE Partition 147	GTR+G	UCE 1200 Right Region, UCE 834 Left Region
UCE Partition 148	GTR+G	UCE 1204 Left Region
UCE Partition 149	GTR+I+G	UCE 518 Core Region, UCE 540 Right Region, UCE 331 Core Region, UCE 1204 Core Region, UCE 1282 Right Region, UCE 359 Left Region
UCE Partition 150	GTR+G	UCE 802 Core Region, UCE 1287 Left Region, UCE 88 Left Region, UCE 759 Left Region, UCE 1209 Left Region
UCE Partition 151	GTR+I+G	UCE 245 Core Region, UCE 1209 Core Region, UCE 283 Right Region, UCE 99 Left Region, UCE 204 Core Region, UCE 25 Left Region
UCE Partition 152	GTR+G	UCE 476 Right Region, UCE 34 Right Region, UCE 1209 Right Region, UCE 747 Core Region
UCE Partition 153	GTR+I+G	UCE 588 Core Region, UCE 1211 Left Region, UCE 696 Core Region, UCE 346 Core Region, UCE 498 Core Region
UCE Partition 154	GTR+I+G	UCE 348 Left Region, UCE 1211 Core Region
UCE Partition 155	GTR+I+G	UCE 1281 Core Region, UCE 1211 Right Region, UCE 660 Right Region
UCE Partition 156	GTR+I+G	UCE 1219 Left Region
UCE Partition 157	GTR+G	UCE 987 Right Region, UCE 466 Right Region, UCE 1219 Core Region
UCE Partition 158	GTR+G	UCE 1219 Right Region, UCE 126 Core Region
UCE Partition 159	GTR+I+G	UCE 1232 Core Region, UCE 305 Core Region, UCE 833 Left Region, UCE 122 Left Region
UCE Partition 160	GTR+G	UCE 122 Core Region
UCE Partition 161	GTR+G	UCE 122 Right Region
UCE Partition 162	GTR+G	UCE 1224 Left Region, UCE 669 Right Region
UCE Partition 163	GTR+G	UCE 1224 Right Region
UCE Partition 164	GTR+G	UCE 1228 Left Region, UCE 690 Right Region, UCE 566 Right Region
UCE Partition 165	GTR+G	UCE 1228 Right Region, UCE 167 Right Region
UCE Partition 166	GTR+G	UCE 1229 Left Region

Partiton Subset	Best Fitting Model	Region(s) in Partition
UCE Partition 167	GTR+G	UCE 1229 Core Region, UCE 966 Core Region
UCE Partition 168	GTR+I+G	UCE 747 Right Region, UCE 711 Core Region, UCE 1229 Right Region, UCE 642 Left Region
UCE Partition 169	GTR+G	UCE 1232 Left Region, UCE 1293 Left Region
UCE Partition 170	GTR+G	UCE 1232 Right Region, UCE 712 Core Region
UCE Partition 171	GTR+G	UCE 1233 Left Region
UCE Partition 172	GTR+I+G	UCE 435 Core Region, UCE 1233 Core Region, UCE 826 Core Region, UCE 891 Core Region
UCE Partition 173	GTR+G	UCE 227 Left Region, UCE 1233 Right Region
UCE Partition 174	GTR+G	UCE 948 Core Region, UCE 516 Core Region, UCE 1234 Core Region, UCE 377 Core Region
UCE Partition 175	GTR+G	UCE 1238 Left Region, UCE 289 Core Region
UCE Partition 176	GTR+G	UCE 1238 Right Region, UCE 549 Right Region, UCE 813 Right Region
UCE Partition 177	GTR+I+G	UCE 458 Left Region, UCE 987 Core Region, UCE 124 Core Region, UCE 792 Right Region, UCE 311 Right Region, UCE 472 Left Region
UCE Partition 178	GTR+G	UCE 1269 Right Region, UCE 1242 Core Region
UCE Partition 179	GTR+I+G	UCE 1242 Right Region, UCE 1253 Right Region
UCE Partition 180	GTR+G	UCE 1243 Left Region, UCE 549 Left Region
UCE Partition 181	GTR+I+G	UCE 1243 Core Region
UCE Partition 182	GTR+I+G	UCE 1243 Right Region, UCE 944 Left Region
UCE Partition 183	GTR+G	UCE 1249 Left Region
UCE Partition 184	GTR+I+G	UCE 674 Core Region, UCE 410 Core Region, UCE 1249 Core Region, UCE 162 Core Region
UCE Partition 185	GTR+G	UCE 1249 Right Region
UCE Partition 186	GTR+I+G	UCE 541 Right Region, UCE 605 Right Region, UCE 1251 Core Region
UCE Partition 187	GTR+G	UCE 690 Left Region, UCE 416 Left Region, UCE 996 Left Region, UCE 749 Core Region, UCE 1296 Right Region, UCE 305 Right Region, UCE 435 Right Region, UCE 1251 Right Region
UCE Partition 188	GTR+I+G	UCE 983 Left Region, UCE 1280 Left Region, UCE 1253 Left Region, UCE 160 Right Region
UCE Partition 189	GTR+G	UCE 858 Left Region, UCE 832 Left Region, UCE 335 Core Region, UCE 1273 Right Region, UCE 348 Core Region, UCE 471 Core Region, UCE 374 Core Region, UCE 305 Left Region, UCE 1256 Left Region, UCE 215 Core Region, UCE 718 Core Region, UCE 525 Right Region
UCE Partition 190	GTR+I+G	UCE 25 Right Region, UCE 1256 Core Region, UCE 459 Left Region, UCE 666 Left Region, UCE 220 Right Region
UCE Partition 191	GTR+I+G	UCE 545 Core Region, UCE 126 Left Region
UCE Partition 192	GTR+G	UCE 73 Right Region, UCE 126 Right Region, UCE 61 Right Region, UCE 983 Core Region, UCE 566 Left Region, UCE 712 Right Region
UCE Partition 193	GTR+G	UCE 545 Right Region, UCE 1260 Left Region
UCE Partition 194	GTR+I+G	UCE 1260 Core Region, UCE 1281 Left Region, UCE 536 Right Region, UCE 835 Left Region, UCE 716 Left Region
UCE Partition 195	GTR+G	UCE 1260 Right Region
UCE Partition 196	GTR+G	UCE 183 Left Region, UCE 674 Left Region, UCE 367 Right Region, UCE 414 Left Region, UCE 971 Left Region, UCE 1263 Right Region
UCE Partition 197	GTR+G	UCE 74 Right Region, UCE 74 Left Region, UCE 1269 Left Region, UCE 416 Right Region, UCE 157 Left Region
UCE Partition 198	GTR+I+G	UCE 982 Core Region, UCE 748 Right Region, UCE 861 Left Region, UCE 408 Right Region, UCE 84 Right Region, UCE 1270 Left Region, UCE 944 Right Region, UCE 136 Right Region, UCE 359 Right Region
UCE Partition 199	GTR+G	UCE 1270 Core Region
UCE Partition 200	GTR+G	UCE 175 Core Region, UCE 191 Right Region, UCE 932 Left Region, UCE 1276 Left Region, UCE 748 Left Region
UCE Partition 201	GTR+I+G	UCE 68 Right Region, UCE 1276 Core Region
UCE Partition 202	GTR+G	UCE 396 Core Region, UCE 1280 Core Region, UCE 356 Core Region, UCE 219 Core Region, UCE 1321 Right Region
UCE Partition 203	GTR+G	UCE 597 Core Region, UCE 394 Left Region, UCE 21 Right Region, UCE 1280 Right Region
UCE Partition 204	GTR	UCE 585 Left Region, UCE 1281 Right Region
UCE Partition 205	GTR+G	UCE 1282 Core Region
UCE Partition 206	GTR+I+G	UCE 152 Core Region, UCE 1286 Core Region, UCE 636 Core Region, UCE 968 Left Region
UCE Partition 207	GTR+G	UCE 640 Left Region, UCE 151 Right Region, UCE 368 Right Region, UCE 1286 Right Region, UCE 592 Core Region
UCE Partition 208	GTR+I+G	UCE 1287 Core Region, UCE 529 Left Region
UCE Partition 209	GTR+G	UCE 129 Left Region
UCE Partition 210	GTR+G	UCE 129 Right Region, UCE 383 Core Region, UCE 750 Left Region
UCE Partition 211	GTR+G	UCE 556 Left Region, UCE 161 Left Region, UCE 815 Right Region, UCE 442 Core Region, UCE 1293 Right Region, UCE 140 Right Region, UCE 1338 Core Region
UCE Partition 212	GTR+G	UCE 1295 Left Region
UCE Partition 213	GTR+I+G	UCE 449 Right Region, UCE 1296 Left Region, UCE 863 Right Region
UCE Partition 214	GTR+G	UCE 1305 Left Region, UCE 527 Left Region
UCE Partition 215	GTR+G	UCE 884 Left Region, UCE 1305 Core Region, UCE 413 Core Region, UCE 525 Left Region
UCE Partition 216	GTR+I+G	UCE 901 Right Region, UCE 921 Core Region, UCE 1305 Right Region
UCE Partition 217	GTR+G	UCE 245 Right Region, UCE 717 Left Region, UCE 179 Right Region, UCE 1307 Left Region, UCE 26 Left Region
UCE Partition 218	GTR+I+G	UCE 332 Core Region, UCE 1307 Core Region, UCE 671 Core Region, UCE 367 Core Region
UCE Partition 219	GTR+G	UCE 1307 Right Region
UCE Partition 220	GTR+I+G	UCE 996 Core Region, UCE 1316 Left Region
UCE Partition 221	GTR+G	UCE 1316 Core Region
UCE Partition 222	GTR+G	UCE 1316 Right Region, UCE 332 Right Region, UCE 665 Right Region, UCE 161 Right Region, UCE 952 Left Region
UCE Partition 223	GTR+G	UCE 1318 Left Region, UCE 469 Right Region, UCE 805 Left Region
UCE Partition 224	GTR	UCE 1318 Core Region
UCE Partition 225	GTR+I+G	UCE 1318 Right Region, UCE 590 Core Region
UCE Partition 226	GTR+G	UCE 1321 Left Region, UCE 84 Left Region, UCE 472 Right Region
UCE Partition 227	GTR+G	UCE 1321 Core Region, UCE 808 Right Region, UCE 957 Left Region
UCE Partition 228	GTR+G	UCE 1324 Left Region, UCE 493 Left Region
UCE Partition 229	GTR+G	UCE 1324 Core Region
UCE Partition 230	GTR+I+G	UCE 1324 Right Region, UCE 377 Left Region, UCE 204 Left Region, UCE 278 Left Region, UCE 717 Right Region
UCE Partition 231	GTR+G	UCE 747 Left Region, UCE 1326 Left Region
UCE Partition 232	GTR	UCE 1326 Core Region
UCE Partition 233	GTR+I+G	UCE 179 Left Region, UCE 1326 Right Region, UCE 346 Right Region, UCE 908 Left Region
UCE Partition 234	GTR+G	UCE 1334 Left Region
UCE Partition 235	GTR+G	UCE 901 Left Region, UCE 568 Left Region, UCE 861 Right Region, UCE 321 Left Region, UCE 1334 Core Region
UCE Partition 236	GTR+G	UCE 787 Core Region, UCE 737 Right Region, UCE 792 Left Region, UCE 1334 Right Region, UCE 466 Left Region
UCE Partition 237	GTR+G	UCE 1338 Right Region, UCE 442 Left Region, UCE 943 Right Region, UCE 712 Left Region, UCE 537 Right Region
UCE Partition 238	GTR	UCE 136 Core Region
UCE Partition 239	GTR+G	UCE 139 Left Region, UCE 238 Core Region, UCE 461 Right Region, UCE 407 Right Region, UCE 910 Core Region
UCE Partition 240	GTR+I+G	UCE 139 Core Region, UCE 276 Left Region
UCE Partition 241	GTR+G	UCE 901 Core Region, UCE 140 Left Region
UCE Partition 242	GTR+I+G	UCE 607 Left Region, UCE 160 Left Region, UCE 525 Core Region, UCE 140 Core Region, UCE 924 Right Region
UCE Partition 243	GTR+G	UCE 142 Left Region
UCE Partition 244	GTR+I+G	UCE 643 Core Region, UCE 142 Core Region, UCE 407 Core Region, UCE 442 Right Region, UCE 883 Core Region, UCE 960 Core Region
UCE Partition 245	GTR+I+G	UCE 142 Right Region
UCE Partition 246	GTR+G	UCE 960 Right Region, UCE 620 Left Region, UCE 143 Right Region, UCE 374 Right Region
UCE Partition 247	GTR+G	UCE 711 Left Region, UCE 149 Right Region, UCE 166 Right Region
UCE Partition 248	GTR+G	UCE 151 Left Region, UCE 980 Left Region

Partiton Subset	Best Fitting Model	Region(s) in Partition
UCE Partition 249	GTR+G	UCE 152 Right Region
UCE Partition 250	GTR+G	UCE 154 Left Region
UCE Partition 251	GTR+I+G	UCE 154 Core Region, UCE 992 Core Region, UCE 805 Right Region, UCE 27 Core Region
UCE Partition 252	GTR+G	UCE 718 Left Region, UCE 154 Right Region, UCE 375 Right Region, UCE 192 Right Region
UCE Partition 253	GTR+I+G	UCE 498 Left Region, UCE 546 Right Region, UCE 159 Left Region
UCE Partition 254	GTR+I+G	UCE 879 Right Region, UCE 160 Core Region, UCE 605 Core Region, UCE 516 Right Region
UCE Partition 255	GTR+G	UCE 166 Left Region, UCE 320 Core Region
UCE Partition 256	GTR+G	UCE 541 Left Region, UCE 167 Core Region
UCE Partition 257	GTR+G	UCE 168 Left Region, UCE 864 Left Region, UCE 870 Right Region
UCE Partition 258	GTR+G	UCE 168 Core Region, UCE 756 Core Region, UCE 762 Right Region
UCE Partition 259	GTR+G	UCE 427 Core Region, UCE 170 Core Region, UCE 826 Right Region, UCE 757 Core Region
UCE Partition 260	GTR+I+G	UCE 971 Core Region, UCE 247 Core Region, UCE 172 Left Region, UCE 749 Left Region
UCE Partition 261	GTR+G	UCE 172 Core Region
UCE Partition 262	GTR+G	UCE 174 Left Region
UCE Partition 263	GTR+G	UCE 174 Right Region, UCE 460 Left Region, UCE 811 Core Region
UCE Partition 264	GTR+G	UCE 176 Right Region, UCE 460 Core Region, UCE 699 Core Region
UCE Partition 265	GTR	UCE 792 Core Region, UCE 179 Core Region
UCE Partition 266	GTR+G	UCE 924 Left Region, UCE 981 Core Region, UCE 474 Core Region, UCE 23 Core Region, UCE 180 Core Region
UCE Partition 267	GTR+G	UCE 194 Right Region, UCE 413 Right Region, UCE 810 Right Region, UCE 180 Right Region
UCE Partition 268	GTR+I+G	UCE 181 Core Region
UCE Partition 269	GTR+G	UCE 185 Left Region
UCE Partition 270	GTR+I+G	UCE 471 Right Region, UCE 185 Core Region
UCE Partition 271	GTR+G	UCE 187 Left Region
UCE Partition 272	GTR+G	UCE 192 Core Region, UCE 376 Core Region, UCE 701 Core Region
UCE Partition 273	GTR+G	UCE 194 Left Region, UCE 863 Left Region, UCE 199 Core Region, UCE 220 Left Region, UCE 787 Right Region, UCE 669 Core Region
UCE Partition 274	GTR+G	UCE 194 Core Region
UCE Partition 275	GTR+G	UCE 824 Right Region, UCE 196 Left Region, UCE 860 Right Region, UCE 54 Core Region, UCE 980 Right Region
UCE Partition 276	GTR+G	UCE 196 Right Region
UCE Partition 277	GTR+G	UCE 981 Right Region, UCE 858 Core Region, UCE 201 Left Region
UCE Partition 278	GTR+I+G	UCE 242 Left Region, UCE 692 Right Region, UCE 797 Core Region, UCE 201 Core Region, UCE 447 Core Region
UCE Partition 279	GTR+G	UCE 201 Right Region, UCE 435 Left Region, UCE 391 Left Region
UCE Partition 280	GTR+G	UCE 203 Core Region
UCE Partition 281	GTR+I+G	UCE 870 Core Region, UCE 203 Right Region
UCE Partition 282	GTR+I+G	UCE 699 Right Region, UCE 569 Right Region, UCE 204 Right Region, UCE 3 Right Region
UCE Partition 283	GTR+G	UCE 21 Core Region
UCE Partition 284	GTR+G	UCE 214 Right Region, UCE 249 Right Region
UCE Partition 285	GTR+I+G	UCE 215 Left Region
UCE Partition 286	GTR+G	UCE 609 Core Region, UCE 215 Right Region, UCE 891 Right Region, UCE 835 Core Region, UCE 351 Left Region
UCE Partition 287	GTR+G	UCE 691 Right Region, UCE 219 Left Region, UCE 830 Left Region, UCE 910 Left Region
UCE Partition 288	GTR	UCE 219 Right Region
UCE Partition 289	GTR	UCE 227 Core Region
UCE Partition 290	GTR+G	UCE 624 Right Region, UCE 227 Right Region, UCE 564 Right Region
UCE Partition 291	GTR+G	UCE 570 Right Region, UCE 23 Left Region
UCE Partition 292	GTR+G	UCE 23 Right Region, UCE 692 Core Region
UCE Partition 293	GTR+G	UCE 231 Left Region
UCE Partition 294	GTR+I+G	UCE 231 Core Region
UCE Partition 295	GTR+I+G	UCE 231 Right Region
UCE Partition 296	GTR+G	UCE 238 Right Region, UCE 411 Left Region
UCE Partition 297	GTR+G	UCE 24 Left Region
UCE Partition 298	GTR+G	UCE 24 Core Region
UCE Partition 299	GTR+G	UCE 846 Core Region, UCE 24 Right Region, UCE 975 Left Region
UCE Partition 300	GTR+G	UCE 242 Core Region
UCE Partition 301	GTR+I+G	UCE 242 Right Region, UCE 624 Left Region
UCE Partition 302	GTR+G	UCE 243 Core Region
UCE Partition 303	GTR+G	UCE 243 Right Region, UCE 27 Left Region, UCE 252 Left Region
UCE Partition 304	GTR+G	UCE 245 Left Region
UCE Partition 305	GTR+I+G	UCE 278 Right Region, UCE 249 Left Region, UCE 924 Core Region, UCE 881 Right Region, UCE 546 Left Region, UCE 504 Core Region, UCE 247 Right Region, UCE 252 Right Region, UCE 665 Core Region
UCE Partition 306	GTR+I+G	UCE 705 Core Region, UCE 394 Core Region, UCE 695 Core Region, UCE 466 Core Region, UCE 249 Core Region
UCE Partition 307	GTR+G	UCE 25 Core Region
UCE Partition 308	GTR+I+G	UCE 750 Core Region, UCE 251 Left Region
UCE Partition 309	GTR+G	UCE 251 Core Region
UCE Partition 310	GTR+G	UCE 251 Right Region
UCE Partition 311	GTR+I+G	UCE 278 Core Region, UCE 26 Core Region, UCE 993 Core Region
UCE Partition 312	GTR+G	UCE 26 Right Region
UCE Partition 313	GTR+G	UCE 267 Core Region, UCE 518 Left Region
UCE Partition 314	GTR+G	UCE 344 Left Region, UCE 267 Right Region, UCE 271 Right Region, UCE 449 Left Region, UCE 344 Right Region
UCE Partition 315	GTR+G	UCE 27 Right Region, UCE 287 Right Region
UCE Partition 316	GTR+I+G	UCE 271 Left Region, UCE 520 Left Region, UCE 468 Left Region, UCE 537 Left Region, UCE 653 Left Region
UCE Partition 317	GTR+I+G	UCE 709 Core Region, UCE 271 Core Region, UCE 446 Core Region, UCE 461 Core Region
UCE Partition 318	GTR+G	UCE 274 Right Region, UCE 592 Left Region, UCE 655 Left Region
UCE Partition 319	GTR+G	UCE 276 Right Region, UCE 836 Left Region
UCE Partition 320	GTR+G	UCE 295 Right Region, UCE 346 Left Region, UCE 280 Left Region
UCE Partition 321	GTR+G	UCE 805 Core Region, UCE 280 Core Region
UCE Partition 322	GTR+I+G	UCE 280 Right Region, UCE 319 Right Region
UCE Partition 323	GTR+G	UCE 282 Right Region, UCE 495 Core Region, UCE 585 Right Region, UCE 731 Core Region, UCE 36 Core Region
UCE Partition 324	GTR+G	UCE 570 Left Region, UCE 283 Left Region
UCE Partition 325	GTR+G	UCE 287 Left Region, UCE 643 Right Region, UCE 695 Left Region, UCE 833 Right Region
UCE Partition 326	GTR+G	UCE 697 Right Region, UCE 287 Core Region, UCE 310 Core Region
UCE Partition 327	GTR+G	UCE 399 Core Region, UCE 288 Left Region, UCE 987 Left Region
UCE Partition 328	GTR+G	UCE 289 Left Region, UCE 709 Left Region
UCE Partition 329	GTR+I+G	UCE 289 Right Region
UCE Partition 330	GTR+I+G	UCE 565 Left Region, UCE 295 Left Region
UCE Partition 331	GTR+I+G	UCE 476 Left Region, UCE 811 Left Region, UCE 836 Core Region, UCE 566 Core Region, UCE 30 Core Region

Partiton Subset	Best Fitting Model	Region(s) in Partition
UCE Partition 332	GTR+I+G	UCE 302 Left Region, UCE 879 Core Region, UCE 734 Right Region
UCE Partition 333	GTR+G	UCE 302 Right Region, UCE 564 Core Region
UCE Partition 334	GTR+G	UCE 307 Left Region
UCE Partition 335	GTR+G	UCE 307 Right Region
UCE Partition 336	GTR+G	UCE 309 Core Region, UCE 367 Left Region
UCE Partition 337	GTR+I+G	UCE 510 Core Region, UCE 309 Right Region
UCE Partition 338	GTR+G	UCE 31 Right Region
UCE Partition 339	GTR+I+G	UCE 310 Right Region
UCE Partition 340	GTR+G	UCE 420 Right Region, UCE 311 Left Region
UCE Partition 341	GTR+I+G	UCE 311 Core Region
UCE Partition 342	GTR+I+G	UCE 42 Left Region, UCE 762 Core Region, UCE 319 Left Region
UCE Partition 343	GTR+I+G	UCE 319 Core Region
UCE Partition 344	GTR+I+G	UCE 320 Right Region
UCE Partition 345	GTR+G	UCE 915 Left Region, UCE 327 Right Region
UCE Partition 346	GTR+G	UCE 331 Left Region
UCE Partition 347	GTR+G	UCE 42 Core Region, UCE 331 Right Region
UCE Partition 348	GTR+G	UCE 332 Left Region
UCE Partition 349	GTR+I+G	UCE 338 Left Region, UCE 84 Core Region
UCE Partition 350	GTR+G	UCE 338 Core Region
UCE Partition 351	GTR+G	UCE 338 Right Region
UCE Partition 352	GTR+G	UCE 34 Left Region, UCE 891 Left Region
UCE Partition 353	GTR+I+G	UCE 351 Right Region, UCE 855 Left Region
UCE Partition 354	GTR	UCE 353 Core Region
UCE Partition 355	GTR+I+G	UCE 356 Left Region, UCE 705 Right Region, UCE 391 Right Region
UCE Partition 356	GTR+G	UCE 948 Right Region, UCE 682 Left Region, UCE 356 Right Region
UCE Partition 357	GTR+G	UCE 358 Right Region
UCE Partition 358	GTR+I+G	UCE 36 Left Region
UCE Partition 359	GTR+G	UCE 36 Right Region, UCE 592 Right Region, UCE 815 Core Region
UCE Partition 360	GTR+I+G	UCE 567 Left Region, UCE 364 Right Region
UCE Partition 361	GTR+I+G	UCE 374 Left Region
UCE Partition 362	GTR+I+G	UCE 375 Left Region
UCE Partition 363	GTR+G	UCE 375 Core Region, UCE 54 Left Region, UCE 797 Left Region
UCE Partition 364	GTR+G	UCE 660 Left Region, UCE 67 Left Region, UCE 376 Right Region, UCE 51 Left Region
UCE Partition 365	GTR+G	UCE 377 Right Region
UCE Partition 366	GTR+G	UCE 38 Left Region
UCE Partition 367	GTR+I+G	UCE 706 Left Region, UCE 383 Right Region, UCE 402 Right Region, UCE 524 Right Region, UCE 858 Right Region
UCE Partition 368	GTR+I+G	UCE 910 Right Region, UCE 392 Left Region
UCE Partition 369	GTR+I+G	UCE 837 Core Region, UCE 980 Core Region, UCE 860 Core Region, UCE 392 Core Region
UCE Partition 370	GTR+G	UCE 396 Left Region
UCE Partition 371	GTR+I+G	UCE 396 Right Region, UCE 532 Core Region
UCE Partition 372	GTR+G	UCE 609 Right Region, UCE 568 Right Region, UCE 399 Right Region
UCE Partition 373	GTR+G	UCE 402 Core Region
UCE Partition 374	GTR+G	UCE 407 Left Region
UCE Partition 375	GTR+G	UCE 408 Core Region
UCE Partition 376	GTR+I+G	UCE 553 Left Region, UCE 410 Right Region, UCE 636 Left Region
UCE Partition 377	GTR+I+G	UCE 413 Left Region, UCE 787 Left Region, UCE 575 Right Region
UCE Partition 378	GTR+G	UCE 572 Right Region, UCE 993 Right Region, UCE 894 Left Region, UCE 414 Right Region
UCE Partition 379	GTR+I+G	UCE 418 Right Region
UCE Partition 380	GTR+G	UCE 42 Right Region, UCE 520 Right Region
UCE Partition 381	GTR+I+G	UCE 74 Core Region, UCE 420 Core Region, UCE 832 Core Region, UCE 834 Core Region
UCE Partition 382	GTR+I+G	UCE 428 Left Region
UCE Partition 383	GTR+G	UCE 428 Core Region
UCE Partition 384	GTR+G	UCE 428 Right Region, UCE 574 Core Region
UCE Partition 385	GTR+G	UCE 431 Left Region
UCE Partition 386	GTR+I+G	UCE 990 Core Region, UCE 585 Core Region, UCE 881 Core Region, UCE 431 Core Region
UCE Partition 387	GTR+G	UCE 759 Right Region, UCE 444 Left Region
UCE Partition 388	GTR+G	UCE 447 Left Region
UCE Partition 389	GTR+G	UCE 447 Right Region, UCE 46 Core Region
UCE Partition 390	GTR+G	UCE 449 Core Region, UCE 693 Right Region
UCE Partition 391	GTR+G	UCE 458 Right Region, UCE 734 Left Region, UCE 693 Left Region
UCE Partition 392	GTR+G	UCE 459 Core Region
UCE Partition 393	GTR	UCE 459 Right Region
UCE Partition 394	GTR+I+G	UCE 561 Right Region, UCE 46 Right Region
UCE Partition 395	GTR+G	UCE 467 Right Region
UCE Partition 396	GTR+I+G	UCE 468 Right Region
UCE Partition 397	GTR+G	UCE 469 Left Region
UCE Partition 398	GTR+G	UCE 469 Core Region
UCE Partition 399	GTR+G	UCE 47 Left Region
UCE Partition 400	GTR+G	UCE 472 Core Region
UCE Partition 401	GTR+G	UCE 474 Left Region
UCE Partition 402	GTR+G	UCE 474 Right Region
UCE Partition 403	GTR+G	UCE 476 Core Region
UCE Partition 404	GTR+G	UCE 761 Core Region, UCE 932 Core Region, UCE 493 Core Region
UCE Partition 405	GTR+I+G	UCE 529 Right Region, UCE 493 Right Region
UCE Partition 406	GTR+I+G	UCE 496 Core Region
UCE Partition 407	GTR+G	UCE 496 Right Region
UCE Partition 408	GTR+G	UCE 498 Right Region
UCE Partition 409	GTR+G	UCE 502 Left Region, UCE 921 Right Region
UCE Partition 410	GTR+G	UCE 502 Core Region
UCE Partition 411	GTR+G	UCE 502 Right Region
UCE Partition 412	GTR+G	UCE 503 Right Region
UCE Partition 413	GTR+G	UCE 504 Left Region
UCE Partition 414	GTR+G	UCE 580 Core Region, UCE 509 Left Region, UCE 716 Right Region

Partiton Subset	Best Fitting Model	Region(s) in Partition
UCE Partition 415	GTR+G	UCE 51 Core Region
UCE Partition 416	GTR+G	UCE 510 Right Region
UCE Partition 417	GTR+G	UCE 516 Left Region
UCE Partition 418	GTR+G	UCE 518 Right Region
UCE Partition 419	GTR+I+G	UCE 61 Core Region, UCE 672 Core Region, UCE 524 Left Region
UCE Partition 420	GTR+G	UCE 524 Core Region
UCE Partition 421	GTR+G	UCE 526 Left Region
UCE Partition 422	GTR+G	UCE 527 Core Region
UCE Partition 423	GTR+G	UCE 529 Core Region
UCE Partition 424	GTR	UCE 536 Core Region
UCE Partition 425	GTR+I+G	UCE 537 Core Region, UCE 759 Core Region
UCE Partition 426	GTR+I+G	UCE 540 Left Region
UCE Partition 427	GTR+I+G	UCE 540 Core Region
UCE Partition 428	GTR+G	UCE 541 Core Region
UCE Partition 429	GTR+G	UCE 545 Left Region
UCE Partition 430	GTR+I+G	UCE 549 Core Region, UCE 88 Core Region, UCE 685 Core Region
UCE Partition 431	GTR+I+G	UCE 552 Left Region, UCE 830 Right Region
UCE Partition 432	GTR+I+G	UCE 552 Core Region
UCE Partition 433	GTR+G	UCE 552 Right Region
UCE Partition 434	GTR+G	UCE 561 Core Region
UCE Partition 435	GTR+G	UCE 564 Left Region
UCE Partition 436	GTR+I+G	UCE 567 Core Region
UCE Partition 437	GTR+G	UCE 567 Right Region
UCE Partition 438	GTR+G	UCE 57 Left Region
UCE Partition 439	GTR+G	UCE 57 Right Region
UCE Partition 440	GTR	UCE 691 Core Region, UCE 572 Core Region
UCE Partition 441	GTR+G	UCE 574 Left Region
UCE Partition 442	GTR+G	UCE 797 Right Region, UCE 952 Right Region, UCE 577 Left Region
UCE Partition 443	GTR+G	UCE 577 Right Region
UCE Partition 444	GTR	UCE 580 Left Region, UCE 943 Left Region
UCE Partition 445	GTR+G	UCE 580 Right Region
UCE Partition 446	GTR+I+G	UCE 588 Left Region
UCE Partition 447	GTR+G	UCE 590 Left Region
UCE Partition 448	GTR+G	UCE 590 Right Region
UCE Partition 449	GTR+G	UCE 597 Left Region
UCE Partition 450	GTR+G	UCE 597 Right Region
UCE Partition 451	GTR+I+G	UCE 598 Left Region
UCE Partition 452	GTR+G	UCE 598 Core Region
UCE Partition 453	GTR+G	UCE 60 Left Region
UCE Partition 454	GTR+G	UCE 60 Right Region
UCE Partition 455	GTR+I+G	UCE 600 Left Region
UCE Partition 456	GTR+I+G	UCE 600 Right Region
UCE Partition 457	GTR+I+G	UCE 607 Core Region
UCE Partition 458	GTR+I+G	UCE 607 Right Region
UCE Partition 459	GTR+G	UCE 61 Left Region, UCE 894 Core Region
UCE Partition 460	GTR+G	UCE 620 Core Region, UCE 768 Core Region
UCE Partition 461	GTR+I+G	UCE 620 Right Region, UCE 727 Left Region
UCE Partition 462	GTR+G	UCE 622 Left Region
UCE Partition 463	GTR+G	UCE 622 Right Region
UCE Partition 464	GTR+I+G	UCE 636 Right Region
UCE Partition 465	GTR+G	UCE 64 Left Region
UCE Partition 466	GTR+G	UCE 64 Right Region, UCE 960 Left Region
UCE Partition 467	GTR+I+G	UCE 640 Right Region, UCE 727 Core Region
UCE Partition 468	GTR+G	UCE 642 Core Region
UCE Partition 469	GTR+I+G	UCE 647 Left Region
UCE Partition 470	GTR+G	UCE 647 Core Region
UCE Partition 471	GTR+G	UCE 648 Left Region, UCE 682 Right Region
UCE Partition 472	GTR+I+G	UCE 648 Right Region
UCE Partition 473	GTR+G	UCE 649 Left Region, UCE 881 Left Region
UCE Partition 474	GTR+G	UCE 649 Right Region
UCE Partition 475	GTR+G	UCE 651 Core Region
UCE Partition 476	GTR+G	UCE 717 Core Region, UCE 651 Right Region
UCE Partition 477	GTR+G	UCE 655 Core Region
UCE Partition 478	GTR+I+G	UCE 745 Left Region, UCE 655 Right Region, UCE 666 Core Region, UCE 908 Core Region
UCE Partition 479	GTR+G	UCE 663 Right Region
UCE Partition 480	GTR+I+G	UCE 830 Core Region, UCE 665 Left Region, UCE 852 Left Region
UCE Partition 481	GTR+I+G	UCE 668 Left Region, UCE 864 Right Region
UCE Partition 482	GTR	UCE 668 Core Region
UCE Partition 483	GTR+I+G	UCE 668 Right Region
UCE Partition 484	GTR+G	UCE 671 Left Region
UCE Partition 485	GTR+I+G	UCE 671 Right Region, UCE 983 Right Region
UCE Partition 486	GTR+G	UCE 672 Left Region, UCE 992 Left Region
UCE Partition 487	GTR+G	UCE 674 Right Region
UCE Partition 488	GTR+G	UCE 777 Core Region, UCE 68 Left Region
UCE Partition 489	GTR+G	UCE 68 Core Region
UCE Partition 490	GTR+I+G	UCE 682 Core Region, UCE 957 Core Region
UCE Partition 491	GTR+I+G	UCE 691 Left Region
UCE Partition 492	GTR+I+G	UCE 696 Left Region
UCE Partition 493	GTR+G	UCE 696 Right Region
UCE Partition 494	GTR+G	UCE 70 Left Region, UCE 943 Core Region, UCE 813 Core Region
UCE Partition 495	GTR+G	UCE 70 Core Region
UCE Partition 496	GTR+I+G	UCE 851 Left Region, UCE 768 Right Region, UCE 70 Right Region
UCE Partition 497	GTR+G	UCE 701 Left Region

Partiton Subset	Best Fitting Model	Region(s) in Partition
UCE Partition 498	GTR+G	UCE 777 Right Region, UCE 701 Right Region
UCE Partition 499	GTR+G	UCE 711 Right Region
UCE Partition 500	GTR+G	UCE 808 Left Region, UCE 716 Core Region
UCE Partition 501	GTR+G	UCE 723 Right Region
UCE Partition 502	GTR+I+G	UCE 727 Right Region
UCE Partition 503	GTR+G	UCE 734 Core Region
UCE Partition 504	GTR+I+G	UCE 736 Core Region, UCE 865 Core Region
UCE Partition 505	GTR+G	UCE 736 Right Region
UCE Partition 506	GTR+G	UCE 745 Core Region
UCE Partition 507	GTR	UCE 750 Right Region, UCE 745 Right Region
UCE Partition 508	GTR+I+G	UCE 756 Left Region
UCE Partition 509	GTR+I+G	UCE 757 Right Region
UCE Partition 510	GTR+I+G	UCE 758 Left Region
UCE Partition 511	GTR+G	UCE 758 Core Region
UCE Partition 512	GTR+I+G	UCE 855 Right Region, UCE 758 Right Region
UCE Partition 513	GTR+G	UCE 76 Core Region, UCE 884 Right Region
UCE Partition 514	GTR+I+G	UCE 76 Right Region
UCE Partition 515	GTR	UCE 781 Left Region
UCE Partition 516	GTR+G	UCE 781 Right Region
UCE Partition 517	GTR+G	UCE 795 Left Region
UCE Partition 518	GTR+G	UCE 795 Right Region
UCE Partition 519	GTR+I+G	UCE 894 Right Region, UCE 98 Core Region, UCE 802 Left Region
UCE Partition 520	GTR+G	UCE 810 Left Region
UCE Partition 521	GTR+G	UCE 810 Core Region, UCE 975 Right Region
UCE Partition 522	GTR+G	UCE 819 Right Region
UCE Partition 523	GTR+G	UCE 824 Left Region
UCE Partition 524	GTR+G	UCE 826 Left Region
UCE Partition 525	GTR+G	UCE 834 Right Region
UCE Partition 526	GTR+I+G	UCE 835 Right Region
UCE Partition 527	GTR+I+G	UCE 836 Right Region
UCE Partition 528	GTR+I+G	UCE 846 Left Region
UCE Partition 529	GTR+G	UCE 846 Right Region
UCE Partition 530	GTR+I+G	UCE 85 Left Region
UCE Partition 531	GTR+G	UCE 85 Core Region
UCE Partition 532	GTR+I+G	UCE 85 Right Region
UCE Partition 533	GTR	UCE 851 Core Region
UCE Partition 534	GTR	UCE 855 Core Region
UCE Partition 535	GTR+G	UCE 863 Core Region
UCE Partition 536	GTR+G	UCE 864 Core Region
UCE Partition 537	GTR+G	UCE 865 Left Region
UCE Partition 538	GTR+I+G	UCE 865 Right Region
UCE Partition 539	GTR+G	UCE 869 Left Region
UCE Partition 540	GTR+G	UCE 869 Core Region, UCE 869 Right Region
UCE Partition 541	GTR+G	UCE 88 Right Region
UCE Partition 542	GTR	UCE 883 Left Region
UCE Partition 543	GTR+G	UCE 883 Right Region
UCE Partition 544	GTR+I+G	UCE 884 Core Region
UCE Partition 545	GTR+G	UCE 89 Core Region
UCE Partition 546	GTR+I+G	UCE 915 Right Region
UCE Partition 547	GTR+G	UCE 921 Left Region
UCE Partition 548	GTR+G	UCE 957 Right Region
UCE Partition 549	GTR+G	UCE 968 Core Region
UCE Partition 550	GTR+G	UCE 971 Right Region
UCE Partition 551	GTR+G	UCE 98 Left Region
UCE Partition 552	GTR+G	UCE 98 Right Region
UCE Partition 553	GTR+G	UCE 997 Left Region, UCE 99 Core Region
UCE Partition 554	GTR+G	UCE 990 Right Region
UCE Partition 555	GTR+I+G	UCE 996 Right Region
Mitochondrial Partition 1	TIM2+F+I+G4	12S, 16S
Mitochondrial Partition 2	TVM+F+I+G4	ATPase6 Codon Position 1, ATPase8 Codon Position 1, ND2 Codon Position 2, ND5 Codon Position 1
Mitochondrial Partition 3	TVM+F+I+G4	ATPase6 Codon Position 2, COIII Codon Position 2, CytB Codon Position 2, ND3 Codon Position 2, ND4 Codon Position 2, ND5 Codon Position 2
Mitochondrial Partition 4	TIM3+F+I+G4	ATPase6 Codon Position 3, ATPase8 Codon Position 3, COI Codon Position 3, COII Codon Position 3, COIII Codon Position 3, ND4L Codon Position 3
Mitochondrial Partition 5	TIM3+F+G4	ATPase8 Codon Position 2, ND2 Codon Position 3
Mitochondrial Partition 6	GTR+F+I+G4	COI Codon Position 1, ND1 Codon Position 1
Mitochondrial Partition 7	GTR+F+I+G4	COI Codon Position 2, COII Codon Position 2, ND1 Codon Position 2, ND4L Codon Position 2
Mitochondrial Partition 8	TVM+F+I+G4	COII Codon Position 1, COIII Codon Position 1, CytB Codon Position 1, ND3 Codon Position 1, ND4 Codon Position 1, ND4L Codon Position 1
Mitochondrial Partition 9	TIM+F+I+G4	CytB Codon Position 3, ND1 Codon Position 3, ND3 Codon Position 3, ND4 Codon Position 3, ND5 Codon Position 3
Mitochondrial Partition 10	TPM3u+F+I+G4	ND2 Codon Position 1
Mitochondrial Partition 11	K3Pu+F+I+G4	ND6 Codon Position 1, ND6 Codon Position 3
Mitochondrial Partition 12	TPM3u+F+I+G4	ND6 Codon Position 2
