# Supplementary material for: Unlocking the genomic potential of historical and formalin-fixed specimens: phylogenetic insights from museum-preserved threadfin fishes (Teleostei: Polynemidae)
Source: PeerJ. 2025 Sep 30;13:e20029. doi: 10.7717/peerj.20029 (PMC13353053; doi:10.7717/peerj.20029)

Polydactylus_bifurcus_raw_AR_Polydactylus_bifurcus_aln_q20_md

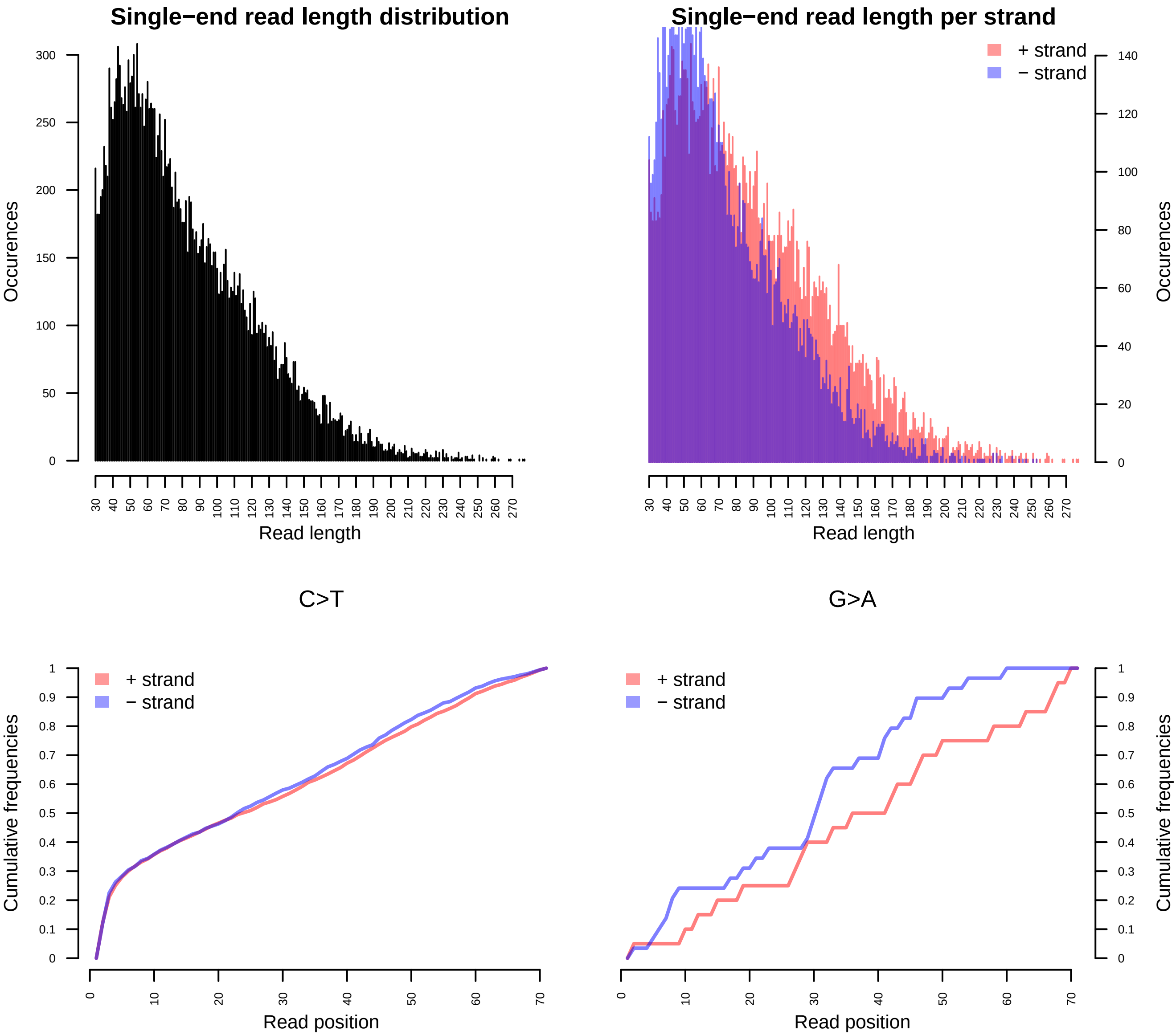

Supplement: Supplemental Information 6 [file peerj-13-20029-s006.zip › bifurcus_mapDamage_ss/Length_plot.pdf]
