# Supplementary figures and images for: Unlocking the genomic potential of historical and formalin-fixed specimens: phylogenetic insights from museum-preserved threadfin fishes (Teleostei: Polynemidae)
Source: PeerJ. 2025 Sep 30;13:e20029. doi: 10.7717/peerj.20029 (PMC13353053; doi:10.7717/peerj.20029)

Polydactylus\_bifurcus\_raw\_AR\_Polydactylus\_bifurcus\_aln\_q20\_md

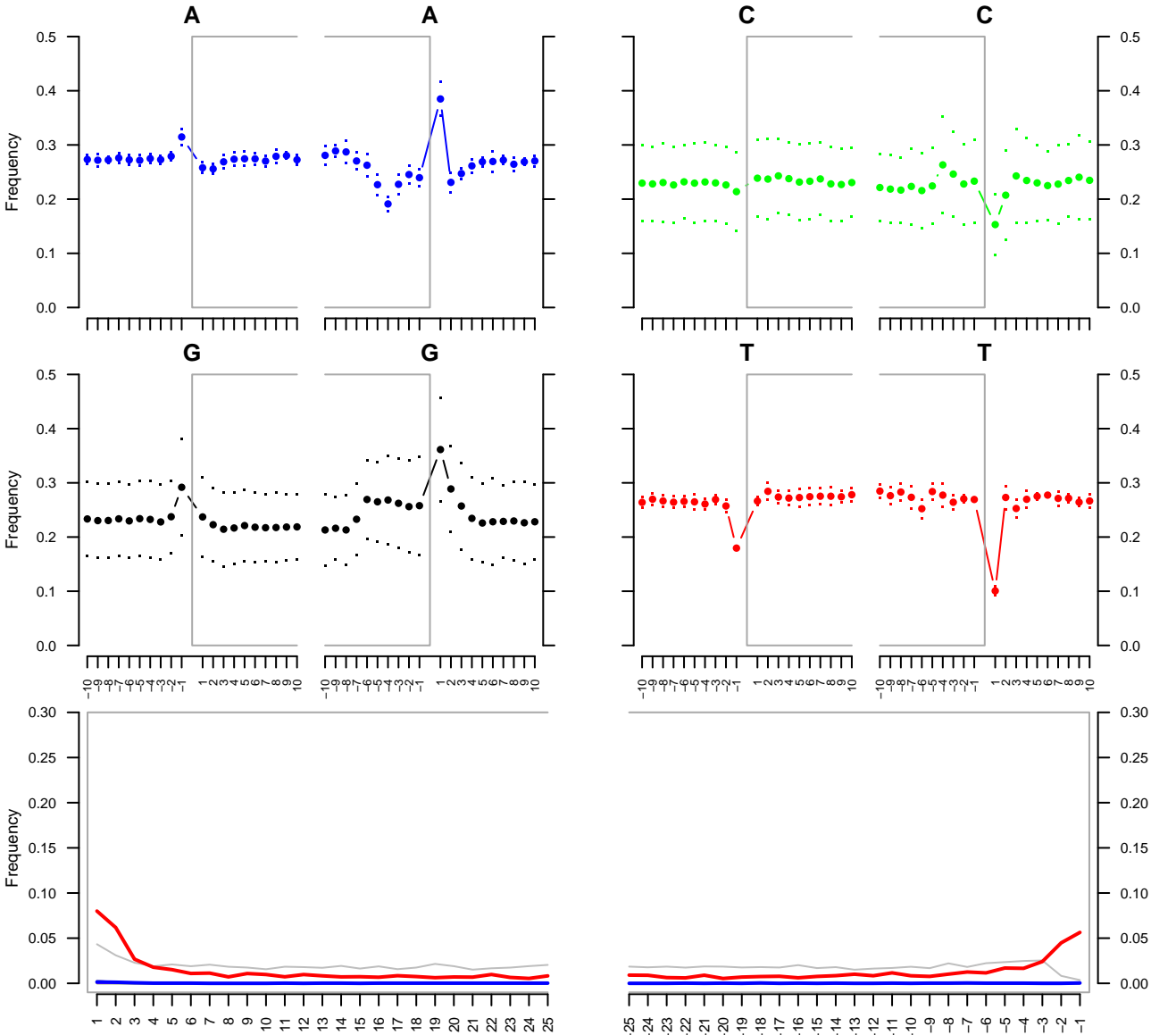

Supplement: Supplemental Information 6 [file peerj-13-20029-s006.zip › bifurcus_mapDamage_ss/Fragmisincorporation_plot.pdf]

ctylus\_macrocephthalmus\_raw\_AR\_Polydactylus\_macrocephthalmus\_aln\_q

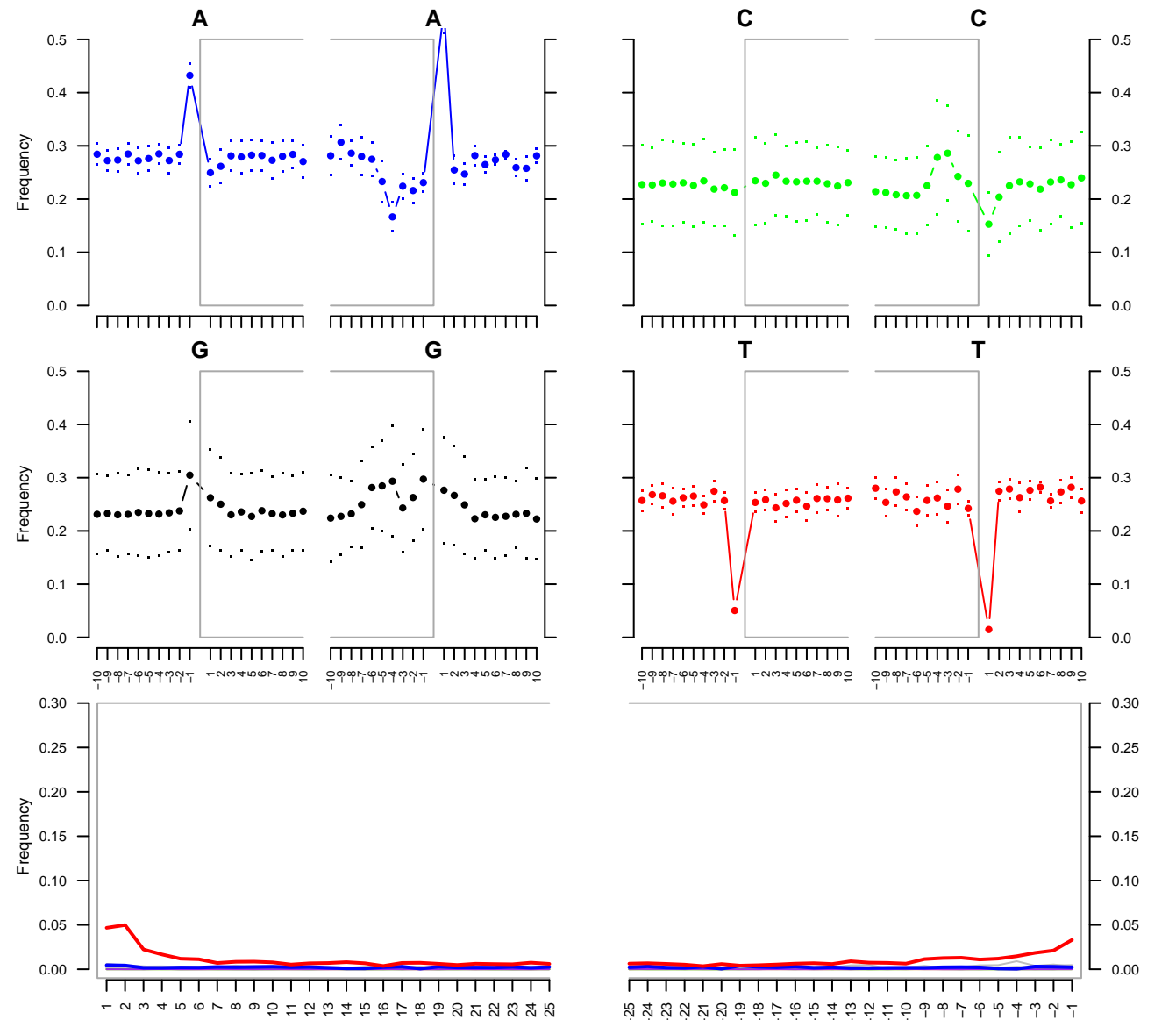

Supplement: Supplemental Information 6 [file peerj-13-20029-s006.zip › macrophthalmus_mapDamage_ss/Fragmisincorporation_plot.pdf]

Single-end read length distribution

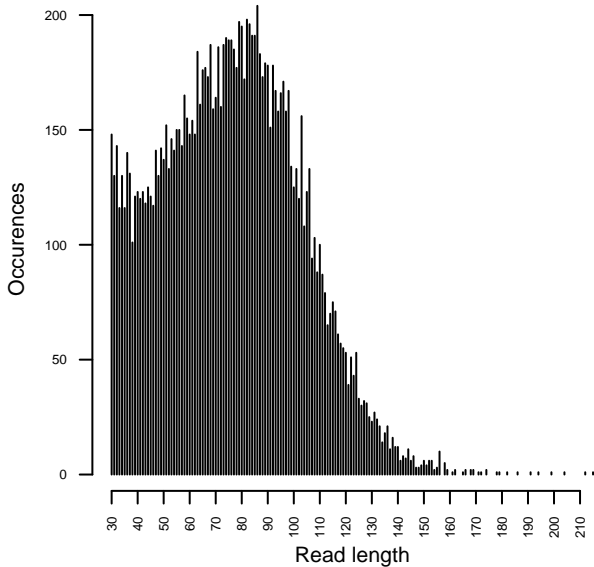

Single-end read length per strand

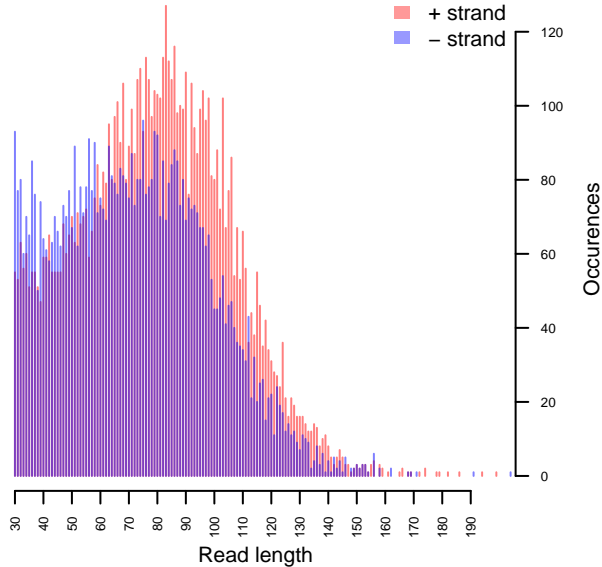

C>T

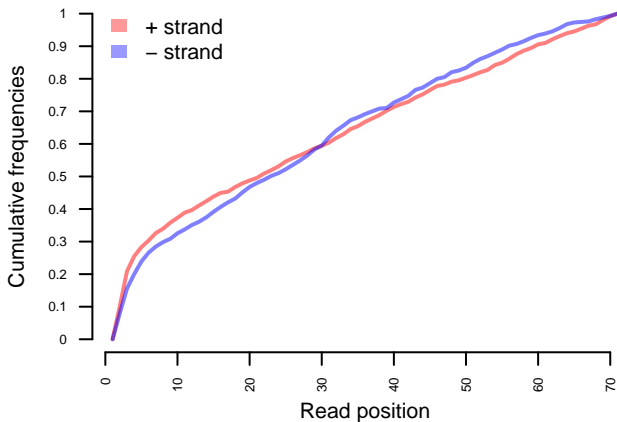

G>A

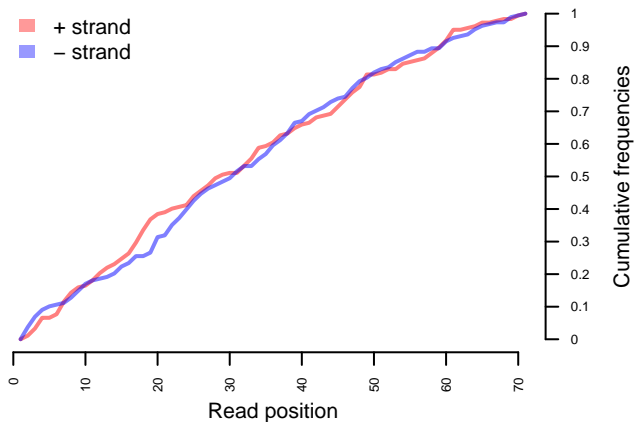

Supplement: Supplemental Information 6 [file peerj-13-20029-s006.zip › macrophthalmus_mapDamage_ss/Length_plot.pdf]
